# Cross-species atlas of bacterial RNA 3′ ends reveals termination mechanistic signatures and evolutionary patterns

**DOI:** 10.1016/j.celrep.2026.118076

**Published:** 2026-10-17

**Authors:** Amir Bar, Yael Altuvia, Michal Eldar, Hanah Margalit

**Affiliations:** 1 Department of Microbiology and Molecular Genetics, IMRIC, Faculty of Medicine, The Hebrew University of Jerusalem, Jerusalem 9112102, Israel

**Keywords:** 3′ terminus, RNAtag-seq, TRS algorithm, transcription termination mechanism, condition-dependent transcription termination, intrinsic terminator, RNase III

## Abstract

Deciphering the generation mechanisms and regulatory roles of bacterial transcript 3′ termini requires large-scale 3′ terminus data. We repurpose PATHOgenex, an available RNAtag-seq dataset, leveraging RNAtag-seq-specific read patterns associated with 3′ termini to generate the “Bacterminome” atlas, a rich resource of species- and condition-dependent transcript 3′ termini of 32 bacterial human pathogens grown under 10–12 conditions. Analysis of sequence elements near 3′ termini revealed, along with the classical intrinsic terminator, other known and newly detected signatures, highlighting the wealth of termination-associated sequence elements. Intriguingly, orthologous genes with corresponding 3′ termini show different termination mechanisms in different bacteria, often matching the dominant mechanism of the respective bacterial species. Analysis of condition-dependent 3′ termini revealed some conservation across species, supporting the conjecture that alternative 3′ termini can play a role in gene expression regulation. Our resource, available through a user-friendly web server (https://bacterminome.huji.ac.il), provides a platform for studying transcription termination at an unprecedented scope.

## Highlights

• “Bacterminome” atlas and web server of bacterial species- and condition-dependent 3′ termini
• Transcript 3′ end generation mechanisms inferred from large-scale 3′ end data alone
• Primary 3′ terminus generation mechanisms are usually more species- than orthologue-specific
• Conserved regulation by condition-dependent premature 3′ termini is detected in orthologues

## Graphical abstract

**Figure:**
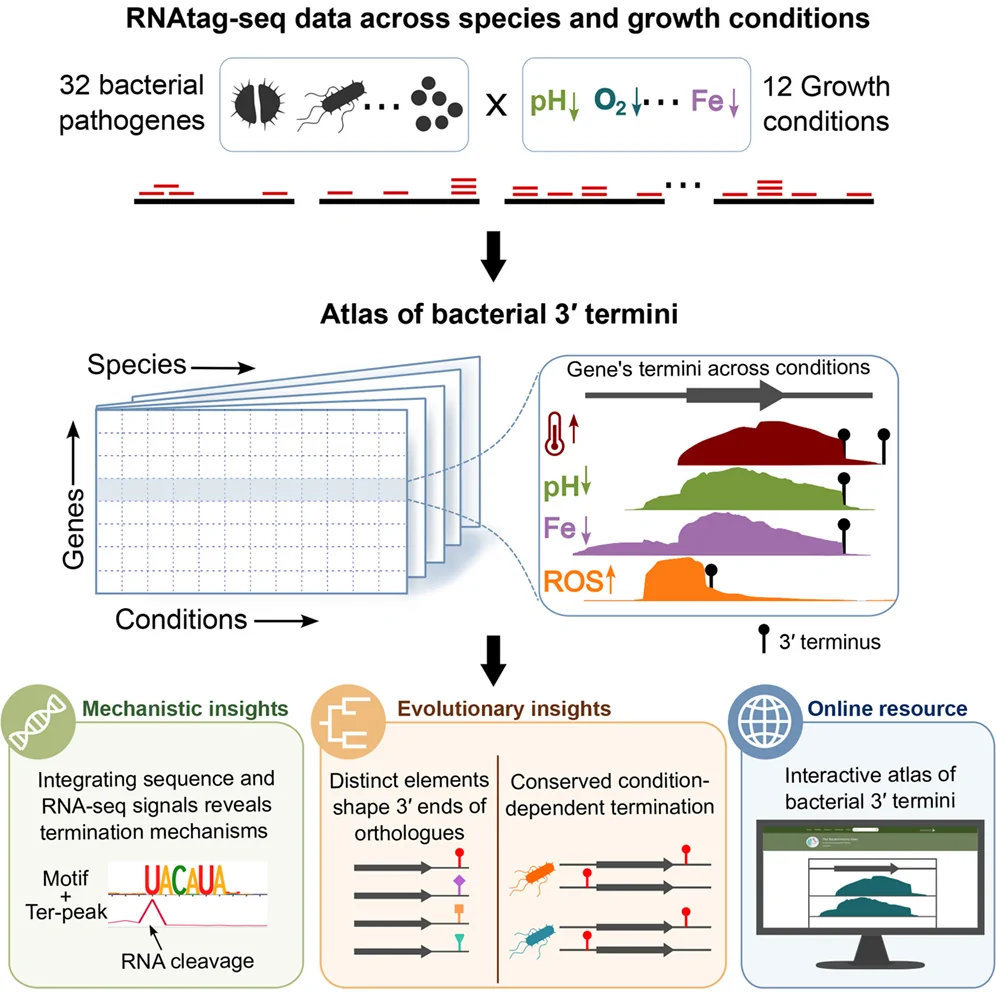

## Teaser

Bar et al. generated a comprehensive atlas of bacterial transcript 3′ termini for 32 bacterial pathogens grown under diverse conditions, enabling comparisons across orthologues and growth conditions. These analyses reveal species-specific elements shaping transcript 3′ ends and conserved regulation by premature termination.

## Introduction

In bacteria, the 3′ terminus of a transcript plays a critical role in shaping RNA stability and, consequently, its expression level. The 3′ end maturation process is dynamic, enabling the cell to exploit it for rapidly adapting gene expression levels in response to environmental cues, such as nutrient availability and stress conditions. This regulation involves various mechanisms, including transcription termination and post-transcriptional cleavage by ribonucleases.

There are two main mechanisms of transcription termination in bacteria: intrinsic termination and termination that requires the ATP-dependent protein factor Rho (Rho-dependent transcription termination).^1^ Intrinsic termination (Rho-independent) involves elements encoded within the sequence of the newly transcribed RNA. The canonical intrinsic terminator involves a GC-rich region that can fold to a stem-loop structure (terminator hairpin), followed by a uracil-rich tract (U-tract),^1^^,^^2^ together promoting the dissociation of the RNA polymerase transcription elongation complex (EC) from the RNA-DNA duplex. In general, this inherent 3′ end stable structure protects the respective transcripts from 3′–5′ exoribonuclease activity.^3^ Consequently, the transcribed transcripts often retain their original 3′ end. In contrast, transcripts terminated by the Rho-dependent mechanism do not necessarily have a stable structure at the termination site. Thus, their 3′ ends are susceptible to cleavage by exoribonucleases, which can digest the originally generated 3′ termini until stopped by a stable structure.^4^^,^^5^ While Rho-dependent and intrinsic transcription termination were mostly considered independent, recent studies suggest they are not mutually exclusive but can, depending on transcript context and organism, operate on the same transcript to reduce termination readthrough and shape transcription termination outcomes.^6^^,^^7^

Transcription termination does not only shape the end of full-length transcripts, but may also play additional regulatory roles.^8^^,^^9^ For example, it can take place at the 5′ untranslated region (UTR) or coding sequence (CDS). In such cases, it is called premature transcription termination. Well-known examples are attenuators^8^ and riboswitches^10^ that sense environmental cues and accordingly alter the RNA structure, either leading to premature transcription termination or enabling transcription to proceed. In addition, primary transcripts generated by both transcription termination mechanisms can be further processed by ribonucleases, resulting in new RNA molecules with new 3′ termini.^11^

To elucidate the roles of these mechanisms in the regulation of gene expression, the 3′ ends of transcripts have to be determined. Initially, prediction tools of 3′ termini were developed (e.g.,^12^^,^^13^^,^^14^^,^^15^^,^^16^). These were based on the detection of features of intrinsic terminators and mainly identified Rho-independent terminators. Subsequently, large-scale approaches, such as term-seq,^17^ Rend-seq,^18^ and SEnd-seq^19^ were developed to experimentally determine the 3′ termini of transcripts. The 3′ termini reported by these methodologies are mainly generated by either intrinsic transcription termination, Rho-dependent transcription termination, or processing by ribonucleases. Application of these approaches has successfully mapped the 3′ termini of transcripts and identified genes in which transcription termination is employed for regulation of gene expression.^17^^,^^18^^,^^20^^,^^21^ In addition, term-seq-derived data of 3′ termini were used to compare properties of Rho-dependent and independent transcript termination sites in *E. coli*^4^ and to explore the interplay between the two mechanisms.^22^ Although powerful, these methodologies have thus far been applied to a limited number of bacterial species and growth conditions, and consequently the currently available data of 3′ termini are insufficient to study transcription termination and/or processing on a wide scale. To overcome this limitation, we recently developed the Termini by Read Starts (TRS) algorithm,^23^ which accurately determines the 3′ termini of transcripts from RNA-seq data based on the RNAtag-seq protocol.^24^ This computational approach is based on our observation that in sequencing results of the RNAtag-seq protocol there is accumulation of reads at 3′ ends of transcripts; identification of such read peaks in RNAtag-seq data enables the determination of 3′ termini. Since RNAtag-seq data are available for many bacterial species and growth conditions, our approach allows a cross-species and cross-condition view of transcript termini and of the 3′ UTRs.

In the current study, we applied TRS to RNAtag-seq data of 32 bacterial pathogens grown under 10 to 12 growth conditions,^25^ and constructed an atlas of 3′ termini across all these species and conditions. This atlas is a rich resource of data, allowing general and comparative analyses of the termini across conditions and species. While numerous 3′ terminus sites at the end of transcripts (Primary termini) were consistent with the canonical intrinsic transcription termination mechanism, we revealed 3′ termini with distinct features, suggesting other species-specific mechanisms for their generation. Furthermore, we found that the 3′ end of orthologous genes is often generated by different molecular mechanisms in different bacterial species. Species-centered analysis revealed that most genes have a single preserved 3′ terminus across conditions. Yet, there is a fraction of genes with condition-dependent alternative 3′ termini, some of which are evolutionarily conserved. This suggests that alternative 3′ termini can be used for gene expression regulation. The atlas of 3′ termini is accessible through a user-friendly web-server, along with tools to visualize and explore the data https://bacterminome.huji.ac.il.

## Results

### Atlas of bacterial 3′ termini

For the construction of the atlas of 3′ termini, we applied TRS^23^ to the PATHOgenex data,^25^ a large-scale transcriptomic dataset generated by the RNAtag-seq methodology^24^ for 32 important human bacterial pathogens grown under 10–12 growth conditions (Figure 1A). These conditions include exponential growth in rich medium as a control and 11 stress conditions that bacteria encounter in the human host upon infection. The species span five bacterial phyla and include both closely related strains (e.g., EPEC and UPEC) and phylogenetically distant bacteria, representing both Gram-negative and Gram-positive lineages. Detailed descriptions of the species and growth conditions are provided in Table S1. For each pathogen grown under a certain growth condition, Avican et al.^25^ prepared three sequencing libraries, for a total of 1,122 sequencing libraries that were available for our analysis. The unmapped read data (FASTA files) were downloaded and subjected to the following multi-step pipeline (Figure 1B and STAR Methods): 1) The read data of each library were processed and mapped to the respective reference genome (Tables S1 and S2). 2) TRS^23^ was applied to the RNA-seq data to determine the 3′ termini of transcripts for each bacterial species and each growth condition (STAR Methods). 3) In parallel, the EggNOG database^26^^,^^27^ was used to determine high quality orthologous gene families. 4) The 3′ termini were assigned to genes based on their genomic annotations (STAR Methods; Figure S1), and, when possible, the assignments were refined based on orthology data (STAR Methods). 5) Finally, combining these data, we obtained a map of 3′ termini for each bacterial species along with an orthology mapping that has enabled the comparison of 3′ termini across bacterial species and growth conditions. This analysis has led to the identification of 107,634 3′ termini across all bacterial species and growth conditions (Table S3A). Since a ribosome depletion step was included in the library construction protocol,^25^ and since for some species several tRNAs are located within rRNA operons, we preferred to omit the 3,167 3′ termini annotated in the vicinity of rRNA and tRNA genes, resulting in a total of 104,467 3′ termini.

**Figure 1:**
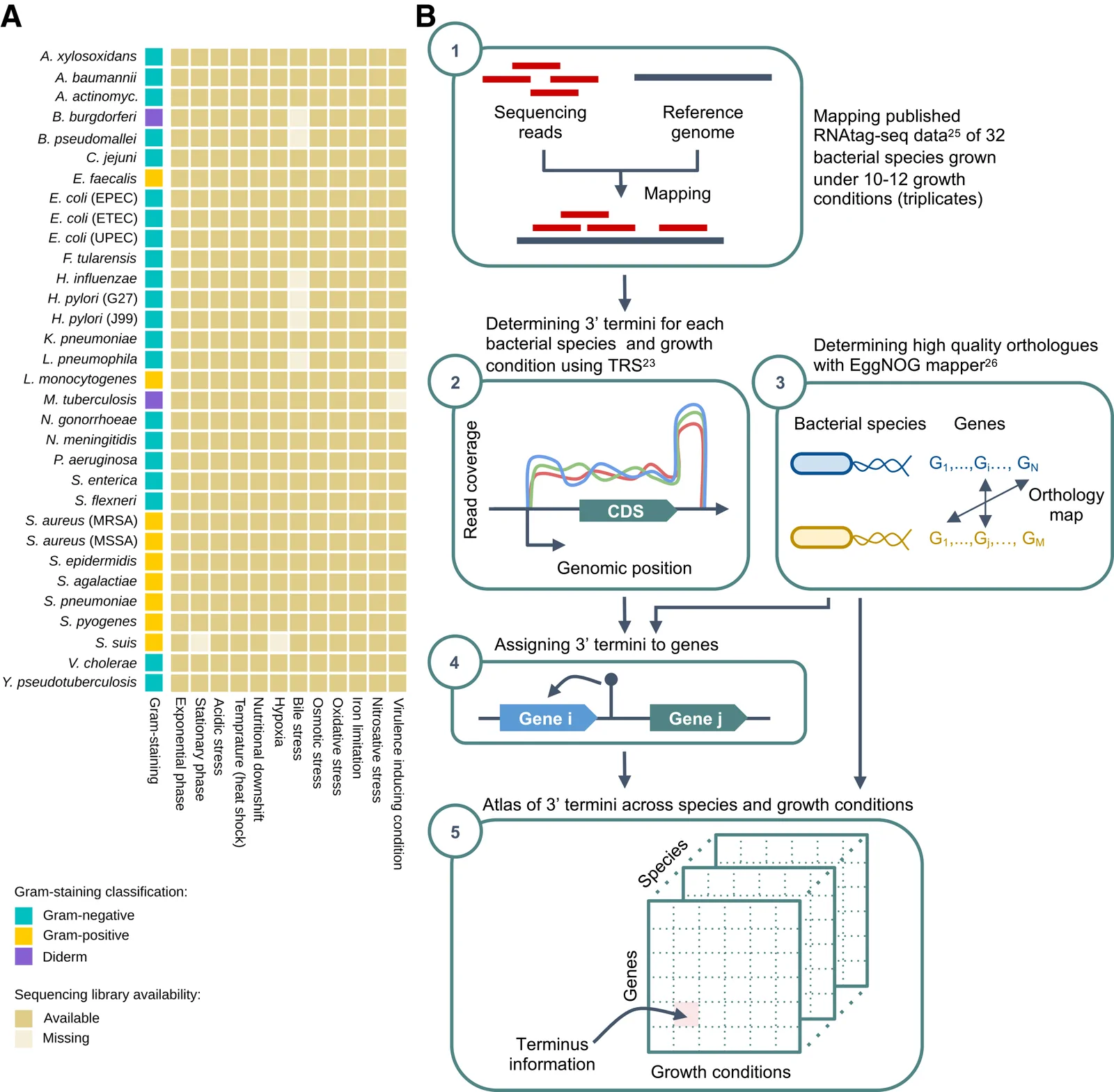
The atlas construction pipeline (A) Presented are the bacterial species and growth conditions in the PATHOgenex dataset.^25^ For each species, its Gram-staining classification and list of growth conditions are indicated. (B) Outlined are the major steps in the construction of the 3′ terminus atlas. For detailed description, see STAR Methods, Figure S1, and Tables S1, S2, and S3.

12,703 (12%) of the 104,467 3′ termini were detected in all growth conditions of the respective bacterial species (10 to 12 conditions), 60,933 (58%) in at least two conditions, and 43,534 3′ termini (42%) in only one condition (Figure 2A). In general, rediscovery of a transcript 3′ terminus in data from multiple growth conditions of a bacterial species supports its reliability. On the other hand, condition-dependent 3′ termini are by definition expected to be detected only in a partial subset of the conditions. Thus, there is an inherent conflict between filtering out falsely detected 3′ termini and retaining the less supported but potentially highly interesting condition-specific ones. Considering the extent and variability of the PATHOgenex data, we took a conservative approach and set for the single-condition 3′ termini stricter thresholds in the TRS analysis (STAR Methods), resulting in the exclusion of nearly 90% of them (Figure 2A). Following this filtering, our final set of 3′ termini included 66,282 sites across all bacterial species. In the final set, 19% are detected in all conditions of the respective bacterial species, and 92% are supported in at least two conditions (Figure 2A; Table S3B).

**Figure 2:**
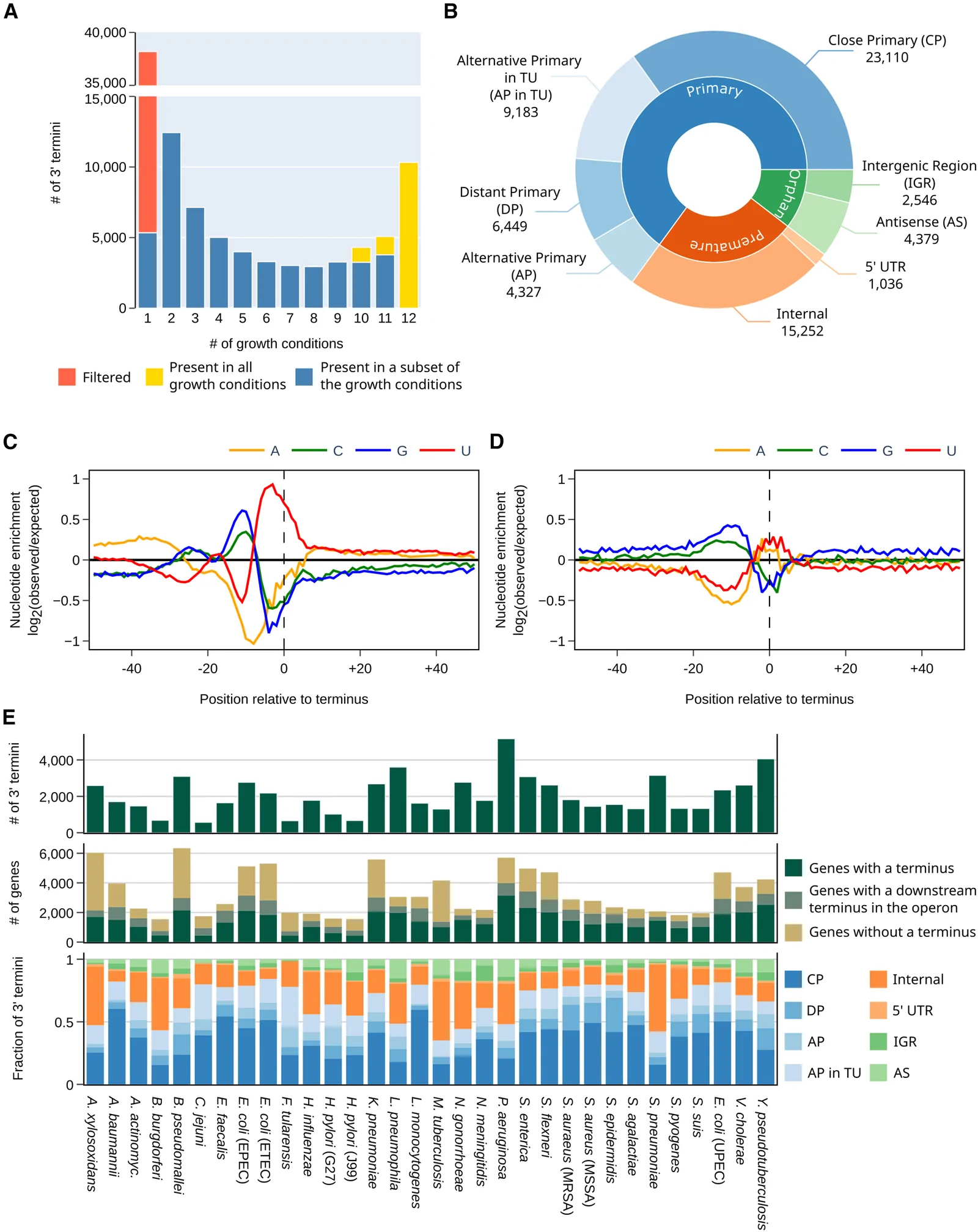
Traits of the 3′ termini in the atlas (A) Reproducibility. For each 3′ terminus site in each bacterial species, the number of growth conditions in which it was identified was counted. Each bar represents the number of 3′ termini that were identified for the respective number of growth conditions indicated on the x axis. In the post-processing steps of the atlas, 38,185 3′ termini detected in only one condition were excluded (red bars), and 66,282 3′ termini remained. Of the remaining 3′ termini, 12,703 termini were identified in all growth conditions (10–12 conditions, considering the maximal number of conditions tested for each bacterial species; yellow bars), further supporting their reliability. (B) Genomic annotations. 3′ termini were assigned three major categories (inner circle) and eight minor sub-categories (outer circle) (Figure S1). The major categories are: (1) Primary – 3′ termini downstream of the stop codon of genes; (2) Premature – 3′ termini internal to the coding sequence or within the gene-estimated 5′ UTR; (3) Orphan – 3′ termini positioned antisense to genes or in intergenic regions. Primary 3′ termini sub-categories include close primary (CP), distant primary (DP), secondary alternative primary (AP), and alternative primary in transcription unit (AP in TU). Non-coding genes were treated similarly to coding genes (STAR Methods). (C and D) Sequence context. Presented is the positional nucleotide enrichment in the flanking 50 nt of the determined 3′ terminus sites. The sequences were aligned by the 3′ terminus positions (labeled 0). The positional nucleotide enrichment was obtained by computing log_2_(observed/expected), where the observed is the actual nucleotide frequency and the expected is the genomic nucleotide frequency. Positional regular average and weighted average of the nucleotide enrichment scores were calculated over all 32 bacterial species (STAR Methods). Shown are the positional weighted averages for Primary (C) and Premature (D) termini. Positional weighted average for Orphan termini and regular average for the three terminus categories are shown in Figure S2. (E) For each bacterial species (x axis), various properties of the 3′ termini were measured and presented in bars. The top image shows the number of 3′ termini identified in each species. The total number of 3′ termini identified is correlated with the individual sequencing library depths (Figure S3) and various genomic factors (Figure S4). Middle image shows the number of annotated genes with a determined 3′ terminus (dark green), the number of genes in an operon that do not have a 3′ terminus, however, there is a primary 3′ terminus downstream in the operon (muted green), or genes without an associated 3′ terminus (mustard). Bottom image shows the number of termini by genomic annotation categories (categories and color-code as in B). For complete terminus and gene details, see Table S3.

### Global traits of the detected 3′ termini

Based on the genomic annotations in the respective bacterial species, the 66,282 3′ termini were classified into the following three major categories: (1) Primary 3′ termini (3′ termini at the end of full operon or gene transcripts, or alternative 3′ termini within operons). (2) Premature 3′ termini (3′ termini located within 5′ UTRs or within the boundaries of annotated genes). (3) Orphan 3′ termini (3′ termini antisense to annotated genes or in intergenic regions) (STAR Methods, Figures 2B and S1). Overall, the group of Primary 3′ termini is the most dominant in our data (65%), followed by Premature 3′ termini (25%) and Orphan 3′ termini (10%). This distribution of 3′ terminus categories is expected given the properties of the TRS algorithm, which is most sensitive to recognizing primary 3′ termini.^23^

To explore the sequence context of the termini, we extracted the genomic sequences flanking each of the 66,282 3′ termini (50 nucleotides upstream and downstream). The sequences were aligned separately for each category of 3′ terminus in each of the 32 bacterial species, using the positions of the 3′ termini as anchors. For each alignment, we calculated a per-position nucleotide enrichment taking into account the individual background nucleotide distribution of the bacterial species analyzed (STAR Methods). Next, we computed for each category of 3′ terminus (i.e., Primary, Premature, and Orphan) the global weighted averages of the positional nucleotide enrichments, using the data of all bacterial species. The strongest signal was observed for the Primary category (Figure 2C), showing two adjacent GC-rich regions followed by a U-rich region, consistent with the sequence profile identified in a similar analysis by Dar and Sorek.^4^ This profile is in accord with the sequence features of the canonical intrinsic terminator (a GC-rich stem loop followed by a U-tract), suggesting that a substantial fraction of the Primary 3′ termini in our data represents canonical intrinsic termination sites at the end of annotated genes or operons. A similar but weaker profile was observed for the Orphan category (Figure S2), suggesting that many of the Orphan 3′ termini represent canonical intrinsic termination sites of yet unannotated genes. The weaker signal is most likely due to the markedly lower number of 3′ termini in this category. Finally, a distinct weak nucleotide enrichment profile was observed for the Premature category (Figure 2D). The profile displayed a GC-rich region followed by a less dominant short AU region, presumably reflecting, not a single, but a collection of stable structures at the 3′ end of premature termination products. Such a 3′ stable structure makes the RNA less susceptible to 3′ end cleavage, enables its accumulation in the cell, and consequently generates a stronger 3′ end signal that could be captured by the TRS algorithm. Overall, the nucleotide enrichment profiles strongly support the reliability of the majority of the determined 3′ termini.

The number of detected 3′ termini per bacterial species varied, ranging from a few hundred (e.g., *F. tularensis* and *C. jejuni*) to thousands (e.g., *Y. pseudotuberculosis* and *P. aeruginosa*) (Figure 2E top). This is also reflected in the variability among bacteria in the number of genes to which 3′ termini were assigned (Figure 2E middle). Several factors likely contribute to this variation, showing statistically significant high correlation with the number of termini (false discovery rate (FDR) adjusted^28^ *p* value ≤0.05): Sequencing depth across libraries (Spearman’s *ρ* > 0.52 for 29 out of the 32 bacterial species and >0.71 for 20 out of the 32; Figure S3), number of genes in the genome, number of transcriptional units in the genome, genome size (length), and GC content (Spearman’s *ρ* = 0.69, 0.73, 0.7, and 0.61, respectively; Figure S4). Notably, the correlation with the genomic-related features differed between Primary and Premature termini (Figure S4). The number of primary termini per a bacterial species strongly correlated with the number of genes, operons, and genome size (Spearman’s *ρ* = 0.8, 0.82, and 0.81, respectively), while GC content had lower correlation (Spearman’s *ρ* = 0.5). Conversely, the number of Premature termini per a bacterial species highly correlated with the GC content (Spearman’s *ρ* = 0.64), while the correlation with each of the other three features was not statistically significant. These differences are conceivable, since every single gene/operon is expected to have at least one functional terminus, while not all transcripts have stable and/or functional premature products. Such products are likely more prevalent in GC-rich transcripts and therefore are more likely to be captured by the TRS algorithm.

Differences between bacterial species are also evident in the distributions of 3′ termini by category (Figure 2E bottom). For example, while in most species the majority of the detected 3′ termini are classified as Primary, in some species, there is a high fraction of Premature 3′ termini. In particular, in four organisms it comprised >40% of the detected termini: *S. pneumoniae* (55%), *M. tuberculosis* (49%), *A. xylosoxidans* (49%) and *B. burgdorferi* (43%). While *A. xylosoxidans* and *M. tuberculosis* are GC-rich, *S. pneumoniae* and *B. burgdorferi* are GC-poor, suggesting that the phenomenon is not fully explained by GC content. In support of our findings, it was recently reported that in *B. burgdorferi*^29^ and *M. tuberculosis*^30^ the majority of termini identified by term-seq were upstream of or internal to the CDS of genes (63% and 68%, respectively). While premature transcription termination or cleavage by an endoribonuclease could be plausible interpretations of this phenomenon, it was recently demonstrated that in *M. tuberculosis* there are many incomplete transcripts due to paused RNA polymerase.^31^

In summary, our data present global as well as species-specific characteristics of the 3′ termini. Whereas some differences between the organisms probably result from technical biases, others likely represent species-specific genomic structures and mechanisms that underlie these differences, hinting at post-transcriptional regulation mechanisms.

### Diverse sequence elements associated with primary 3′ termini

Following previous studies showing an association between sequence elements and generation of 3′ termini (e.g.^32^^,^^33^), we searched independently in each bacterial species for sequence motifs in the 3′ UTRs of genes (defined by the Primary terminus; STAR Methods). This analysis revealed a total of 340 motifs (Table S4), some of which could be associated with previously annotated sequence elements (Table S5). The latter included elements that were reported to be linked with transcription termination and/or RNA processing. Clustering the 340 motifs (STAR Methods; Table S4) produced 216 motif clusters, 84 of which (accounting for nearly 60% of all detected motifs) could be divided into 42 pairs of complementary motifs. Since the respective complementary motifs often resided on the same 3′ UTR, this phenomenon probably reflects a wealth of stem structures in the 3′ UTRs. Most clusters (175) were species-specific, and only 41 were detected in at least two bacterial species, hereinafter termed multi-species clusters. Yet, the multi-species clusters accounted for nearly half (49%) of all detected motifs. As expected, the largest multi-species cluster corresponded to a uridine stretch (U-motif), a hallmark of the canonical intrinsic terminators. This motif was found in 23 out of 32 bacterial species. The second largest multi-species cluster corresponded to an adenine stretch (A-motif), which was identified in 14 species. Consistent with the Primary terminus enrichment profile and with previous reports,^1^^,^^34^ these two motifs frequently co-occurred, where the A-motif was typically identified at a close distance upstream of the U-motif.

While the motif search and clustering revealed recurrent sequence elements across bacterial species, they did not establish whether or how these motifs contribute to the generation of Primary 3′ termini. To better assess the relationship between a motif and a terminus, we calculated the median and median absolute deviation (MAD) of motif distances from the 3′ terminus (STAR Methods). Additionally, we returned to the raw read start counts (corresponding to 3′ ends of sequenced fragments) to generate a per-position score for 3′ terminus signals across all sequencing libraries of a given species (hereinafter, ter-peak score; STAR Methods). The median and MAD highlight motif proximity to the 3′ terminus and positional consistency, and the ter-peak score provides a refined view of where the terminus signal peaks relative to the motif. Applying these measures to the U-motif across species revealed a median distance of 1–3 nucleotides (nts) from the 3′ terminus, with the peak signal usually located between the 6^th^ and 8^th^ nucleotides of the stretch (Figure 3A), consistent with the known mode of canonical intrinsic terminators.^1^ Since the U-motif represents a well-established sequence element directly involved in transcription termination, these observations support the utility of motif proximity as an indicator of association with the generation of 3′ termini. Thus, we heuristically applied a threshold of median≤15 to identify motifs that are highly likely associated with the generation of primary 3′ termini, termed hereinafter pTAMs (primary Terminus Associated Motifs). Overall, 67 pTAMs were identified across all species. Interestingly, when we restricted the motif clusters to pTAMs, only seven clusters were multi-species clusters. Apart from the U-motif and A-motif clusters, the only additional pTAM cluster spanning more than two closely related species contained a Repetitive Extragenic Palindromic (REP) element (Table S5), identified in five *Enterobacteriaceae* species.

**Figure 3:**
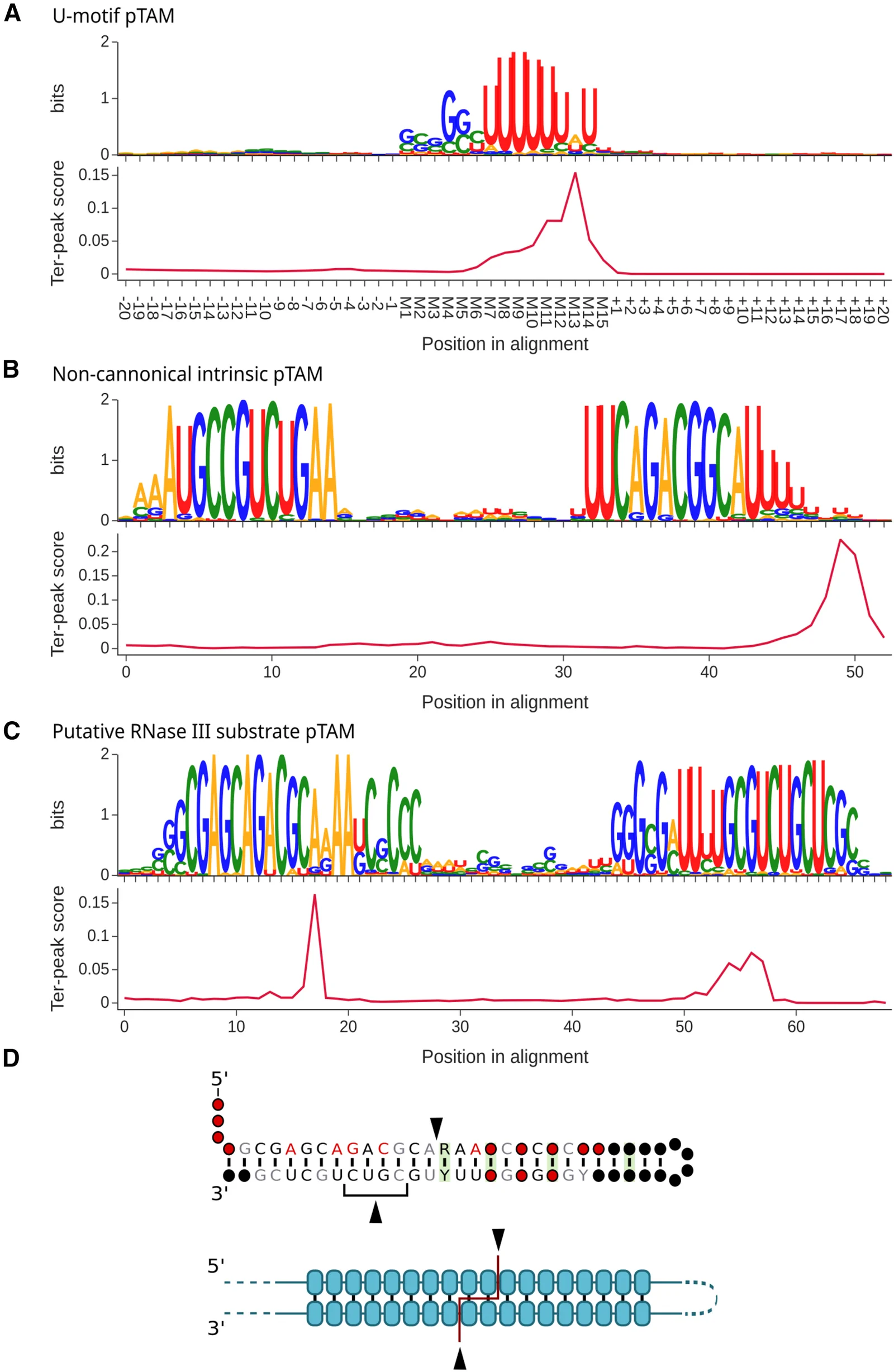
Sequence motifs associated with Primary 3′ termini (A–C) Examples of pTAMs with their flanking regions, shown in a logo format and their positional ter-peak score (upper and lower images, respectively; STAR Methods). Note that ter-peak scores are not scaled between motifs. (A) U-motif in *S. enterica* (identified in 383 out of 1,791 3′ UTR sequences); (B) DNA uptake sequence motif as part of a non-canonical intrinsic terminator in *N. gonorrhoeae* (based on 416 out of 983 sequences); and (C) Tuberculosis Rho-Independent terminator (TRIT) motif in *M. tuberculosis* (based on 72 out of 377 sequences). (D) Upper image: The predicted structure of the TRIT element based on the multiple sequence alignment of the identified motif sites (STAR Methods). Letter codes R, Y, and circles indicate purine, pyrimidine, and any nucleotide, respectively. Letter colors indicate nucleotide identity percentage: 75% (gray), 90% (black), or 97% (red). Circle colors indicate the percentage of nucleotide presence (as opposed to indel) in the sequence-structure alignment: 75% (gray), 90% (black), or 97% (red). Arrows indicate the positions with high ter-peak scores. Green shading indicates covariation between nucleotides to preserve base pairing. Lower image: a schematic of the RNase III substrate and cleavage pattern. See also Figure S5.

Overall, these results suggest that while U-rich motifs associated with intrinsic termination are widespread across bacterial species, many motifs associated with Primary 3′ termini are restricted to specific bacterial lineages.

### Species-specific sequence and structure elements in the 3′ UTR shape the 3′ terminus

While the U-motif was the most widespread pTAM across bacterial species, it was not always the dominant pTAM within a given species. In several bacteria, other motifs were more prevalent, suggesting that diverse sequence elements contribute to the generation and processing of Primary 3′ termini. To better understand this diversity, we manually inspected the individual pTAM sequences and their respective ter-peak values, resulting in the characterization of four major pTAM classes: (1) Canonical intrinsic terminators – pTAMs in this class showed the above-described U-motif, which was typically preceded by a GC stretch with low information content that could potentially generate the stem structure. (2) Non-canonical intrinsic terminators – pTAMs in this class resembled an intrinsic terminator, with a sequence that folds into a stem, albeit not the GC-rich canonical sequence. The most pronounced example was a motif matching a DNA uptake sequence in both *N. meningitidis* and *N. gonorrhoeae*.^35^ Interestingly, the motif co-occurred with a reverse complement motif and in most cases was followed by a U-tract (Figure 3B). Indeed, it was previously reported that DNA uptake sequences were co-opted to serve as intrinsic terminators in these species.^15^^,^^33^ Additional motifs in our data had similar properties; i.e., they showed high information content, and the sequence could fold into a non-canonical stem followed by a short U-tract. Such motifs were classified as non-canonical intrinsic terminators. (3) Putative substrates of RNase III cleavage – We identified five pTAMs that suggested the involvement of RNA processing events. Two pTAMs matched the Correia Repeat (CR) in *N. meningitides* and *N. gonorrhoeae*^36^ and three co-occurring pTAMs matched the Tuberculosis Rho-Independent terminator (TRIT) in *M. tuberculosis*^14^ (Figures 3C and S5). To support the hypothesis of processed termini, we independently analyzed these pTAMs as follows: we extracted for each gene the full sequence of the reported respective element spanning the pTAM regions, aligned them, and computed the ter-peak score along the alignment (STAR Methods). In the CR element, the strongest signals coincided with previously reported RNase III cleavage sites,^37^^,^^38^^,^^39^ supporting the interpretation that the CR element undergoes RNase III-mediated processing (Figure S5). The TRIT element exhibited two high ter-peak scores (Figure 3C). Viewing the ter-peak scores along the predicted RNA secondary structures (STAR Methods), we observed a putative dual-site cleavage pattern, reminiscent of processing by RNase III^40^^,^^41^^,^^42^ (Figure 3D). While the regulatory role and underlying mechanism of TRIT have yet to be fully elucidated, our results strongly suggest it is involved in shaping the transcript 3′ terminus, presumably via RNase III cleavage. (4) Unclassified elements – This class comprises pTAMs for which no underlying mechanism could be assigned. The most prominent example of this group is the above-described REP element that was classified as pTAM in five Enterobacteriaceae species. Indeed, REP elements were previously discussed in the context of transcription termination and protection from exonuclease activity (e.g.^4^^,^^5^^,^^43^).

Having classified pTAMs into four groups (Figures 4A–4D), we next assessed how these classes are utilized across bacterial species. To this end, for each bacterial species, each gene with a pTAM was assigned to one of the above classes. Genes with a Primary terminus without an associated motif were classified by the presence of a nearby U-tract, as an estimator of intrinsic termination (STAR Methods; Figure 5A). Note that while there are examples of genes in which the 3′ end is shaped by multiple mechanisms,^22^^,^^45^ for simplicity we related to the most evident termination-associated element. We observed the dominance of motifs related to intrinsic termination, reinforcing the predominance of the intrinsic termination mechanism in bacterial cells. Still, there was a variation in the type and relative fraction of elements used by individual bacterial species to shape their 3′ terminus (Figure 5B). The most pronounced example is in *M. tuberculosis*, for which we barely identified elements associated with intrinsic termination. Consistent with our results, *M. tuberculosis* was indeed recently reported to mostly utilize Rho-dependent termination, with only rare occurrences of intrinsic terminators.^30^^,^^46^ Intriguingly, we found multiple examples of orthologous genes that use different sequence and structure elements to shape their 3′ UTRs (Table S4A). For example, for *sucD*, encoding succinate coenzyme A ligase, we observed four distinct classes of 3′ terminus-associated elements across species: a canonical intrinsic terminator, a non-canonical intrinsic terminator (DNA uptake element), a 3′ terminus within a TRIT element (presumably due to cleavage by RNase III), and an unclassified structure lacking a U-tract (Figure 4E). Another example is *cysE*, encoding serine acetyltransferase (Figure S6A). Interestingly, differences were observed even in closely related bacterial species. For example, the sequences downstream of the CDS of *cysE* in *N. gonorrhoeae* and *N. meningitidis* are highly similar except for an insertion of a CR element in *N. meningitidis*. Accordingly, the CR-related 3′ terminus site of *cysE* is observed only in *N. meningitidis* (Figure S6B). These examples suggest that elements associated with the 3′ terminus can differ between orthologous genes, implying different termination mechanisms. In summary, our analyses reveal that while intrinsic termination-associated elements dominate across bacteria, species differ markedly in the repertoire of sequence and structure elements used to shape transcript 3′ termini.

**Figure 4:**
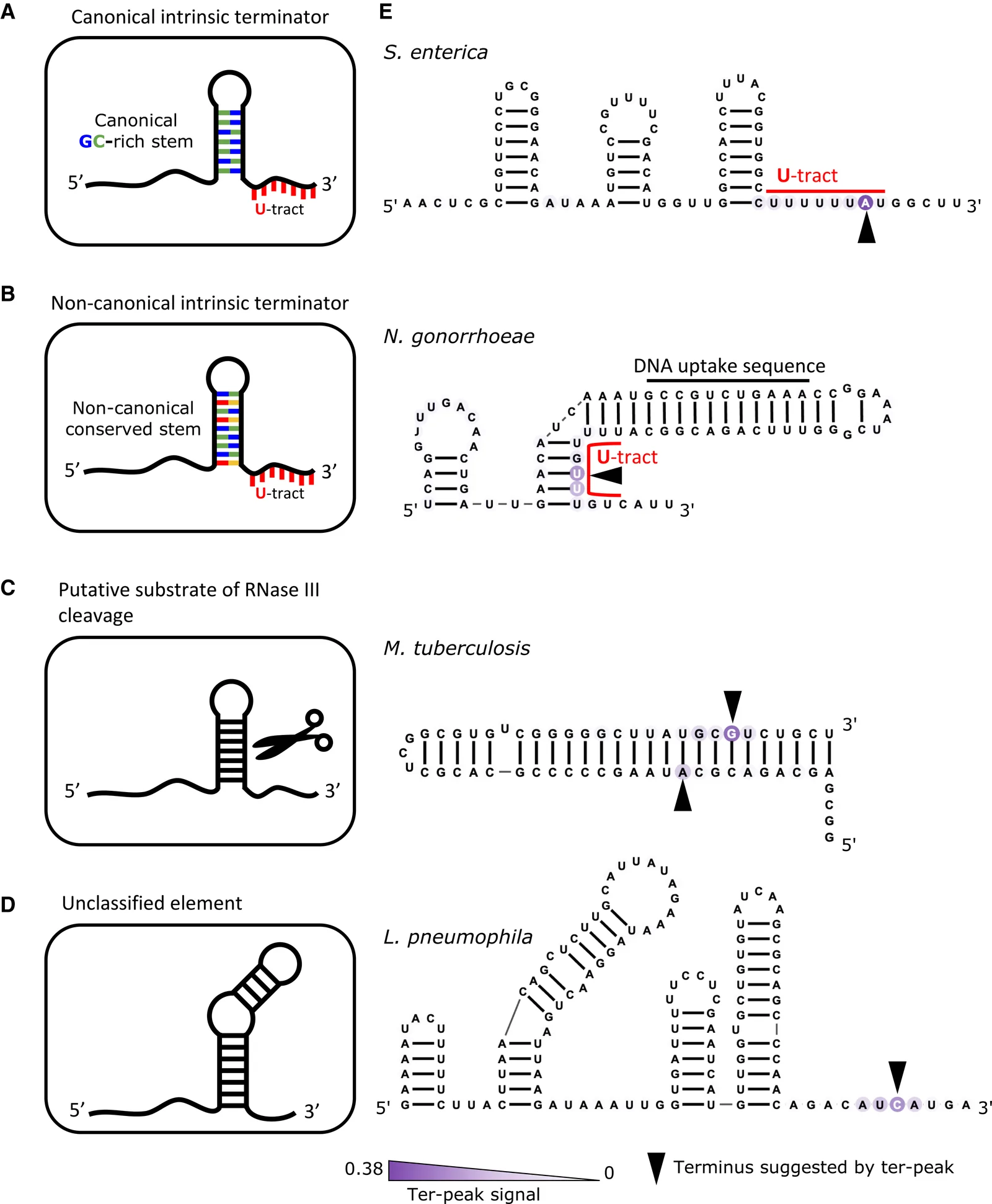
Sequence and structure elements shaping a 3′ terminus (A–D) Presented in boxes are the four sequence-structure elements observed in the vicinity of the Primary 3′ termini. (A) Canonical intrinsic terminator composed of a GC-rich stem followed by a U-tract. (B) Non-canonical intrinsic terminator composed of a unique sequence that can fold into a stem structure followed by a U-tract. (C) Putative RNase III substrate – specifically the CR^36^ and the TRIT^14^ elements. (D) Unclassified element. (E) For each sequence-structure element, shown to the right are the predicted structures of the 3′ UTR in orthologues of the *sucD* gene (STAR Methods), demonstrating the different elements shaping the 3′ terminus of the same gene in different species. The 3′ terminus, as suggested by the ter-peak, is marked with an arrowhead. The 3′ UTR sequence is colored by the 3′ UTR ter-peak score at each position, with darker purple signifying a higher ter-peak score. See also Figure S6; Tables S4 and S5.

**Figure 5:**
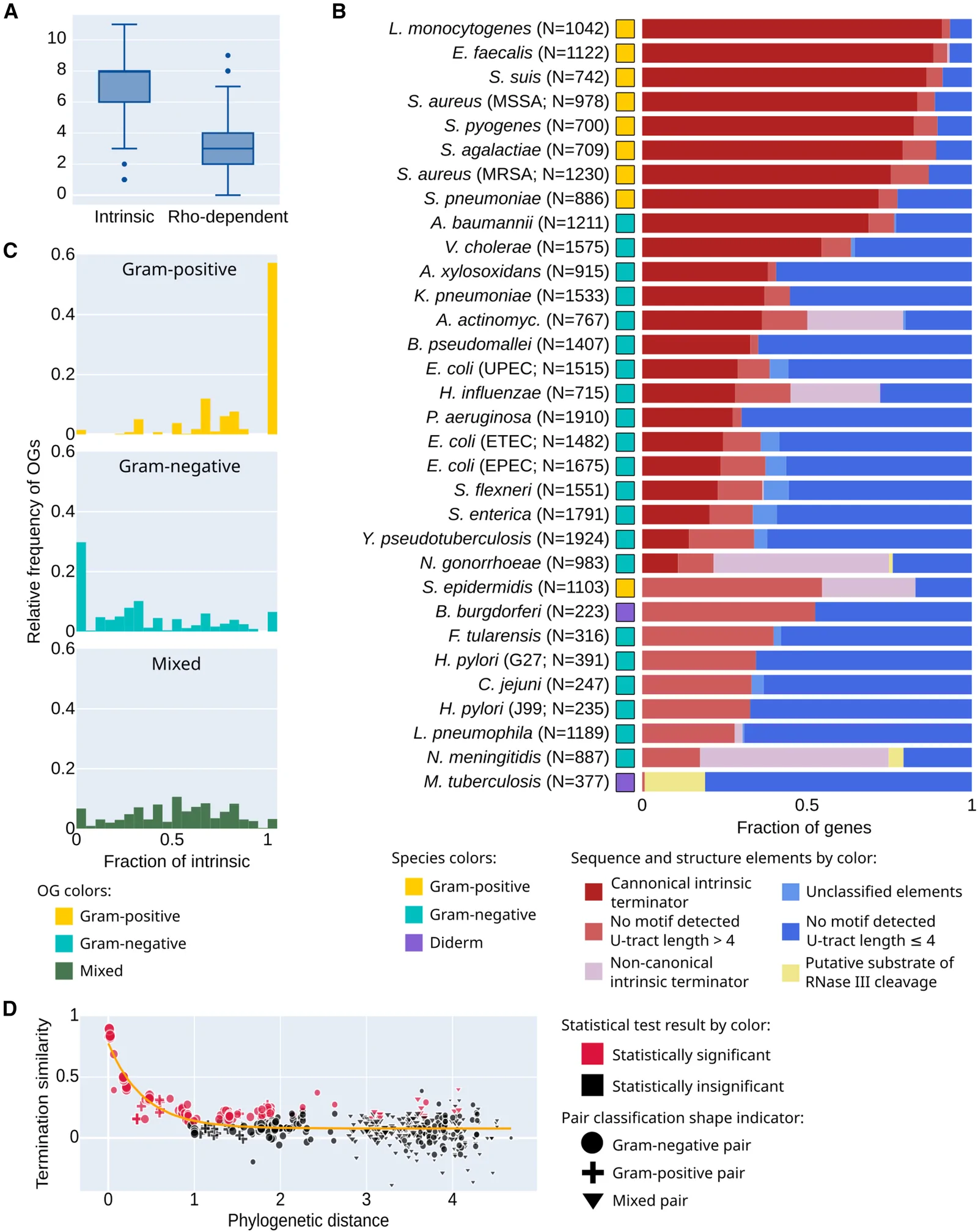
Utilization of sequence and structure elements in the vicinity of Primary 3′ termini by the various species (A) The distribution of the U-tract lengths of Rho-independent terminators (Intrinsic) and Rho-dependent terminators in *E. coli* based on termini classified to either mechanism by Dar and Sorek^4^ (STAR Methods). Most Rho-dependent terminators have a U-tract of length ≤ 4 nt, thus we use a threshold of five to estimate Intrinsic termination utilization. (B) Classification of genes by sequence and structure elements in the vicinity of Primary 3′ termini. For each bacterial species (y axis), the bars present the fractions of genes associated with each group of elements. Genes were first categorized by the four 3′ terminus-associated elements: (1) Canonical intrinsic terminator (dark red); (2) non-canonical intrinsic terminator (pink); (3) putative substrate of RNase III cleavage (yellow); and (4) unclassified elements (light blue). The remaining genes for which we failed to find an element were categorized by their U-tract length as described in A: putative intrinsic – U-tract length > 4 (light red) or putative non-intrinsic – U-tract length ≤4 (dark blue). Squares to the right of the species names are color-coded according to the Gram-staining group: Gram-positive (yellow), Gram-negative (teal), or diderm (bacteria with two cell membranes that fall outside conventional Gram-stain classification; purple). The three distributions differed statistically significantly (*p* value = 1.04E-141 in Kruskal-Wallis test, followed by Dunn’s test, *p* value ≤ 3.49E-30 in all pairwise comparisons). Overall, Gram-positive species in our data show a high tendency toward intrinsic terminators. (C) For each OG containing more than two species, we calculated the fraction of intrinsically terminated genes. The histograms show the distributions of the utilization of intrinsic terminators across OGs unique to Gram-positive species (top image), Gram-negative species (middle image), and OGs spanning both Gram categories (termed “mixed”; bottom image). (D) Scatterplot of the similarity score computed by the Matthews correlation coefficient^44^ (MCC), as a function of the phylogenetic distance (see also Figure S7). Species pairs are marked as follows: plus (both are Gram-positive), circle (both are Gram-negative), and triangle otherwise (mixed pairs or both diderm). Symbol size reflects the number of orthologous genes with a detected Primary terminus in both species. The statistical significance of a similarity score was assessed by applying a one-sided permutation test with 100,000 shuffles. Scores were considered statistically significant if their *p* value was below 0.05 after FDR correction.^28^ Symbols are colored red if the similarity score passed the statistical test and black otherwise. Orange lines indicate the regression fit of an exponential decay model (STAR Methods).

### Primary 3′ terminus-associated elements may vary between orthologous genes

Next, we sought to systematically assess the phenomenon of species-specific termination mechanisms for orthologous genes, analyzing all sets of orthologous genes with a Primary terminus in our data. To simplify cross-species comparisons of termination mechanisms, we reduced our detailed classification of genes into a binary scheme based solely on their U-tract length (Figure 5A; STAR Methods). In this scheme, genes were classified as either “intrinsic” (U-tract length > 4) or “alternative” (mechanisms that are not intrinsic termination; U-tract length ≤4). We used these classifications in three analyses at different resolutions to examine how conserved the use of the termination mechanism is across species.

Initially, we examined whether termination mechanisms are conserved within orthologous groups (OGs). To prevent bias from pairs of closely related species, we included only OGs present in at least three species and calculated for each OG the fraction of genes classified as intrinsic. Since Gram-positive species were underrepresented and displayed higher fractions of genes associated with intrinsic termination compared to Gram-negative species (Figure 5B), we grouped OGs based on their Gram-staining: OGs restricted to Gram-positives (*N* = 393), OGs restricted to Gram-negatives (*N* = 1,372), and the remaining OGs, hereinafter mixed OGs (*N* = 743). We observed that the utilization of intrinsic terminators statistically significantly differed between the three groups (Figure 5C; *p* value = 1.04E-141 in Kruskal-Wallis test, followed by Dunn’s test, *p* value ≤ 3.49E-30 in all pairwise comparisons), where OGs restricted to Gram-positives and Gram-negatives showed skewed distribution toward intrinsic and alternative mechanisms, respectively. By contrast, “mixed” OGs showed a weaker tendency toward a specific mechanism. The distributions of the fractions of genes in each OG that are classified as intrinsic are non-degenerate, indicating that the termination mechanism of orthologous genes is not strictly homogeneous even within OG restricted to a specific Gram group. Moreover, the wide variability of the mixed group distribution may imply that the termination mechanism is less conserved in orthologous genes of phylogenetically distant species. Still, a subset of 74 mixed OGs (10%) consistently retained the same termination mechanism across species.

Since OGs within each Gram group showed a tendency toward a specific termination mechanism, we next asked whether similarity in termination mechanisms follows phylogenetic relationships. To this end, we first generated a phylogenetic tree of the 32 bacterial species (STAR Methods; Figure S7A). Next, for each pair of species, we restricted the data to the subset of OGs with classified termini in both species and represented each species as a vector with binary values (intrinsic or alternative). Similarity in termination mechanisms between a pair of species was quantified as the fraction of genes that were identically classified. This similarity decreased gradually with increasing phylogenetic distance (STAR Methods; Figure S7B). Some distant species pairs, involving at least one Gram-positive species, showed high similarity (Figure S7B). However, this similarity diminished when using Matthews correlation coefficient^44^ (MCC) to account for the imbalance in the ratio of genes classified with intrinsic and alternative termination mechanisms in each species (Figure 5D). This suggests that this similarity is mostly attributed to the relatively high usage of intrinsic termination in these species rather than conservation of gene-specific mechanisms.

Finally, we asked whether termination mechanisms are primarily gene-driven (inherited by orthologues) or species-driven. To this end, for each species, we divided the genes into two groups, orthologous and species-specific (genes without an orthologous gene in our data), and compared the frequency distributions of intrinsically and alternatively terminated genes between the two groups. Only three species (*B. Pseudomallei*, *P. aeruginosa*, *and V. cholerae*) showed a statistically significant difference (*ꭕ*^2^ test, adjusted *p* values ≤ 9.35E-03 after Bonferroni correction), consistent with the conjecture that termination mechanism preferences reflect the species tendencies.

Taken together, while there is a non-negligible subset of genes for which the termination mechanism is conserved across all species, our analyses indicate that termination mechanisms generally align with species-level characteristics rather than orthologous gene identity.

### Internal motifs associated with 3′ termini

To identify motifs associated with Internal 3′ termini, we extracted for each bacterial species and each Internal 3′ terminus the genomic sequences flanking the Internal 3′ terminus (±25 nt). Sequence motifs were independently searched for each bacterial species (STAR Methods). The number of detected motifs per species ranged from zero to six, summing to a total of 50 across all species (Table S6). Unlike Primary 3′ termini, we did not find a dominant motif common to many species. Since these motifs were searched in the vicinity of the 3′ end to begin with, we further filtered them by their ter-peak score and not by their distance from the 3′ end (STAR Methods). This resulted in 22 internal Terminus Associated Motifs (iTAMs) spanning 10 bacterial species. In five species, we found 16 iTAMs that could be associated with mobile genetic elements. Most of these iTAMs occurred in fewer than 20 genes and/or within genes with multiple conserved copies. Some of these iTAMs co-occurred and comprised a longer element. In contrast, in three species, *L. monocytogenes*, *S. aureus* MRSA252, and *S. aureus* MSSA476, we identified an abundant short motif. This motif matched the recognition site of the MazF toxin of the MazEF toxin-antitoxin system.^47^^,^^48^^,^^49^^,^^50^ The MazF toxin was identified in various bacteria, including *L. monocytogenes*, *S. aureus* MRSA252, and *S. aureus* MSSA476, where it has been implicated in stress-response regulation.^51^^,^^52^^,^^53^ We not only detected the motif but also identified the expected cleavage site within the motif (Figure 6A). These findings firmly suggest that our data capture genuine MazF cleavage events in multiple genes of these three species.

**Figure 6:**
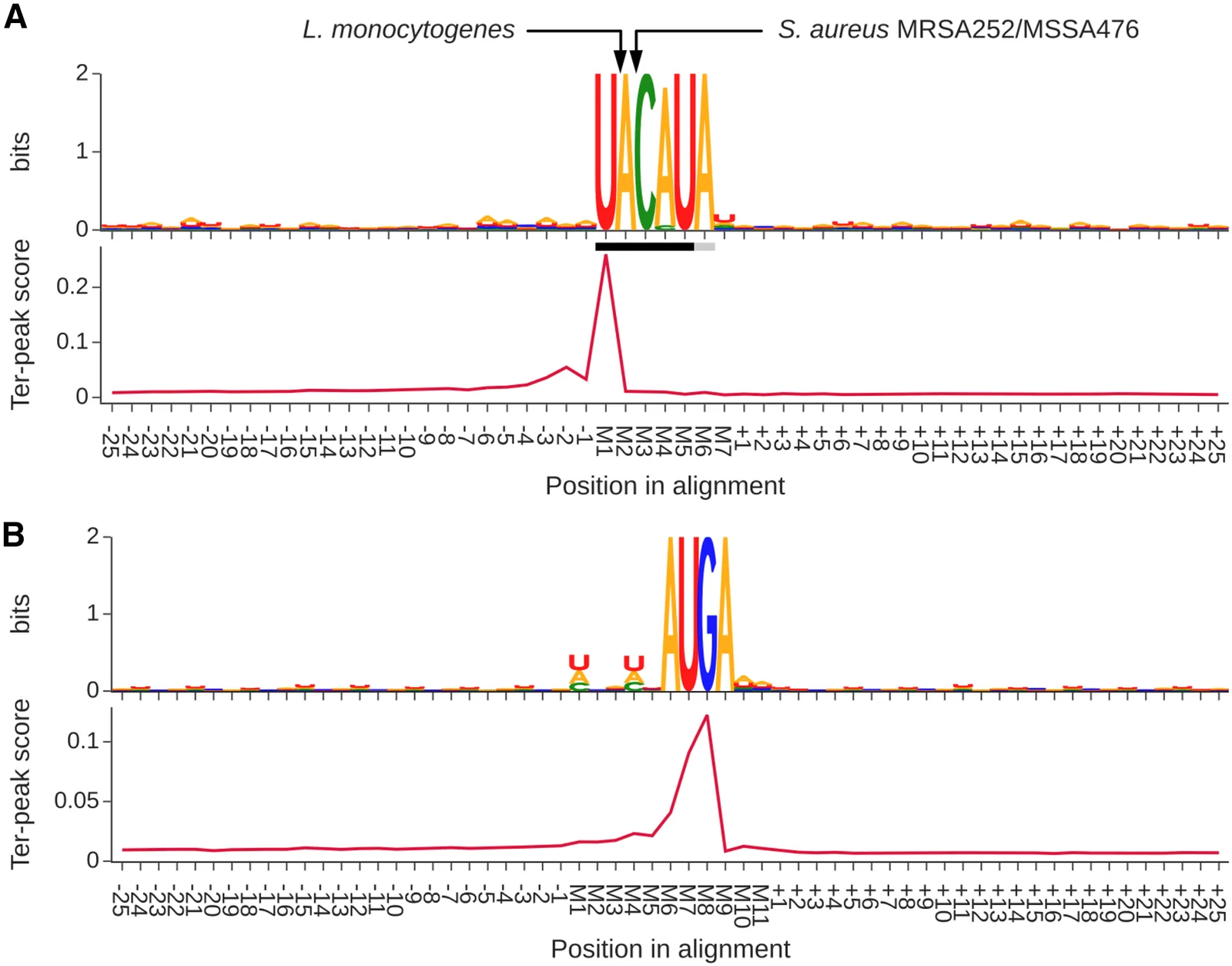
iTAM examples Shown are logo presentations of two sequence motifs detected in the vicinity of Internal termini along with their flanking regions (STAR Methods). (A) The *L. monocytogenes* iTAM matching the MazF recognition sequence (identified in 107 out of 235 input sequences). The motif is underlined by black and gray lines matching the shorter, widely acknowledged MazEF motif (e.g.,^48^^,^^49^^,^^50^), and the one-nucleotide extension we found, respectively. Interestingly, the exact same “A” extension was recently reported for *B. subtilis*.^47^ The ter-peak signal in the motif region suggests that the cleavage occurs between “U” and “A” (left black arrow). For comparison, the ter-peak-derived cleavage site for *S. aureus* MRSA252 and *S. aureus* MSSA476 strains is also depicted (right black arrow). Both cleavage modes have been previously reported in various species (e.g.,^50^^,^^54^^,^^55^). However, our derived cleavage site for the *S. aureus* strains is one nucleotide downstream of the previously reported site.^50^ (B) The *S. pneumonia* AUGA iTAM (identified in 614 out of 1607 input sequences). The highest ter-peak score was obtained between “G” and “A”, where “G” is the last nucleotide of the transcript.

Another intriguing short, highly abundant internal motif was found in *S. pneumoniae*, where 38% of the sequences displayed a strong 5′-AUGA-3′ signal. The highest ter-peak score was obtained between “G” and “A” (Figure 6B). Although AUGA has been associated with translational coupling between adjacent genes, this mechanism is unlikely to explain our observation, as these motifs were not dominantly identified near gene boundaries. Instead, the ter-peak score peaked within the motif, resembling the ter-peak score pattern observed for MazF and suggesting possible cleavage by a yet unidentified toxin. Further studies are needed to determine the biological role of this motif.

### Evolutionarily conserved condition-dependent regulation by alternative 3′ termini

In a previous study, we exemplified that TRS applied to RNAtag-seq data enables identification of condition-dependent 3′ termini that are used for regulating gene expression.^23^ Here, we leverage our extensive multi-species dataset comprising diverse growth conditions to investigate whether condition-dependent regulation of transcription termination is conserved across bacterial species. To this end, we first evaluated the number of 3′ terminus sites associated with each gene in each species regardless of its growth conditions (Figure 7A). We observed that, in all species, the majority of genes (ranging between 56% and 92%) were associated with a single 3′ terminus across the growth conditions. An exception was *S. pneumoniae*, in which 57% of the genes were associated with two or more termini. To identify genes with conserved condition-dependent 3′ termini, we analyzed the distribution of raw read start counts, corresponding to transcript 3′ ends, along each gene. The cumulative distribution function (CDF) of these read starts reflects the positional distribution of termination events: when premature termination occurs, the majority of reads accumulate upstream of the premature terminus, whereas in the absence of premature termination, reads are distributed further along the gene, reaching the Primary terminus (Figure 7B). Leveraging this property, we quantified shifts in the termination site by calculating, for each gene exhibiting both Primary and Premature termini, the difference in CDF values at the Premature terminus across pairs of growth conditions. This difference serves as a between-condition score, reflecting the relative dominance of premature versus primary termination in one of the compared conditions. The maximal score across all condition pairs was taken as the gene’s final score. We computed these scores for each gene with both Primary and Premature termini in every species and compared scores of orthologous genes (STAR Methods), identifying 47 unique candidates with conserved condition-dependent termination site shifts (Table S7A). Many of the orthologous genes with conserved condition-dependent regulation (18 unique genes) were identified in pairs of Enterobacteriaceae species, likely reflecting the high representation of this family in our dataset. Gene Ontology (GO) analysis of these genes (STAR Methods), revealed enrichment for 10 slim GO terms, associated with metabolic processes, translation-related functions, and responses to stimuli (Table S7B). Indeed, there are known examples in these categories of genes regulated by premature transcription termination-related mechanisms, although not necessarily in the exact same species or growth conditions for which we found the condition-dependent termination (e.g., *tyrS*,^56^ *ribB*,^57^ and *suhB*^58^). Interestingly, typical read coverage patterns of known condition-dependent regulated genes (Figure 7C) were also identified for additional candidates. The most prominent was the carbonic anhydrase gene *yrdA* in EPEC, displaying regulation by an alternative 3′ terminus. Under exponential growth, the full-length transcript terminating at the primary site is observed. However, under hypoxic conditions, full-length transcription is suppressed, and read accumulation at the presumptive 5′ UTR of *yrdA* suggests premature termination (Figure 7D). Remarkably, *S. enterica* exhibits similar read pattern differences between exponential and hypoxic conditions (Figure 7D), although the premature 3′ termini occur at different positions, located within the 5′ UTR in EPEC and within the coding sequence in *S. enterica*. These results provide proof of concept that our dataset enables the identification of condition-dependent regulation by alternative 3′ termini conserved across species, and demonstrate its potential to detect additional such candidates.

**Figure 7:**
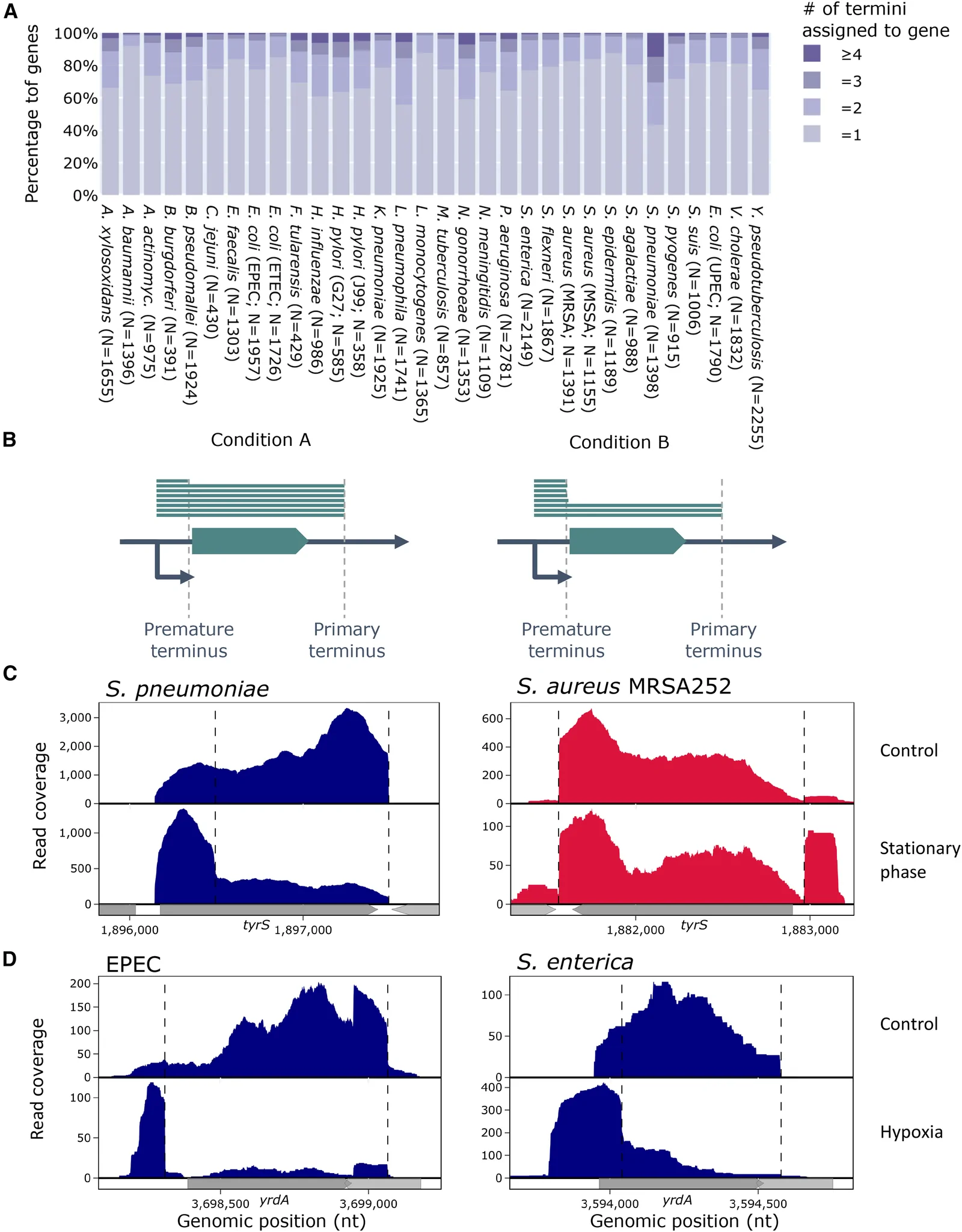
Analysis of condition-dependent termini (A) For each gene, we calculated the number of its associated 3′ termini. This number was used to classify the genes into four groups having 1, 2, 3, and ≥4 associated 3′ termini (colored from light to dark purple, respectively). Presented for each species are the proportions of these groups. (B) Illustration of the distribution of sequenced fragments in Primary versus Premature termini in two different conditions. In condition A, most sequenced fragments end at the Primary 3′ terminus, whereas in condition B most sequenced fragments end at the premature 3′ terminus. (C and D) Read coverage along the genes *tyrS* (tyrosine tRNA ligase) in *S. pneumoniae* and *S. aureus* MRSA252 (C) and *yrdA* in *S. enterica* and EPEC (D) in two conditions demonstrating potential regulation by a Premature 3′ terminus. The read coverage color indicates the strand of the transcribed gene: forward (blue), reverse (red). Positions of 3′ termini associated with the genes are indicated by a black dashed line. Note that read coverage (y axis) is not scaled across libraries and species.

## Discussion

In this study, we utilized available gene expression data to computationally determine the 3′ terminus sites of genes in 32 human bacterial pathogens grown under various growth conditions, resulting in an atlas of over 60,000 sites. To the best of our knowledge, this is the first large-scale mapping of condition-dependent 3′ termini for most species in our data, providing a rich resource for studying mechanistic, regulatory, and evolutionary aspects of the termination sites.

While our atlas provides a rich resource of 3′ termini, it is likely incomplete, even under the growth conditions tested. First, it was constructed by applying TRS^23^ to RNAtag-seq^24^ data^25^ rather than using a dedicated 3′ end mapping approach (e.g., term-seq,^17^ SEnd-seq^19^). In a previous study we compared the 3′ termini obtained by TRS applied to RNAtag-seq with those obtained by term-seq and demonstrated high precision, but the recall was approximately 70% of the total 3′ termini, with most of the missing sites corresponding to premature termination events.^23^ Therefore, here also premature sites may be underestimated. Secondly, the PATHOgenex protocol filtered RNA fragments shorter than 100 nt,^25^ excluding short products of premature transcription termination, processing intermediates, and small non-coding RNAs. These constraints, together with the inherent instability of prematurely terminated and processed transcripts, contribute to an underrepresentation of transient RNA products generated by early termination or processing. Despite these limitations, the inclusion of numerous growth conditions provides strong support to many termini that were reproducibly identified across multiple conditions, suggesting that the most robust sites are well captured. To further enhance transparency and utility, we provide a user-friendly web server that enables researchers to visualize read coverage and read starts and assess the data the 3′ termini are based on (https://bacterminome.huji.ac.il). Thus, although the atlas may not encompass the full complexity of bacterial Terminomes (the repertoire of 3′ termini), it offers a sound foundation for comparative analyses and hypothesis generation at an unprecedented scale.

The above limitations are critical to consider when evaluating our finding that most genes were associated with a single 3′ terminus, as this may reflect detection constraints rather than the absence of condition-dependent regulation. It is likely that such regulatory events are more widespread than our results suggest, particularly in operonic contexts, where an alternative terminus may occur within an upstream gene. In such cases, two genes could each appear to have a single 3′ terminus, yet still exhibit condition-dependent regulation at the operon level. However, we intentionally refrained from operon-level analyses since they require accurate transcription unit annotations within species, and defining orthologous operons across species is not trivial. Nevertheless, even our relatively coarse, gene-level heuristic approach identified examples of condition-dependent regulation that are conserved across species, suggesting that the dataset likely contains more such events awaiting discovery. This resource thus provides a valuable foundation for future, more detailed studies to reveal additional layers of bacterial gene expression regulation.

It is noteworthy that detailed molecular insights can be derived de-novo from routinely generated RNA-seq datasets, genomic annotations, and a reference genome. By integrating sequence information with 3′ end signals, we were able to reaffirm and refine several known observations, including the cleavage mode of MazF, the processing of CR elements, and the adaptation of DNA uptake sequences as intrinsic terminators. At the same time, we expanded the knowledge concerning the TRIT element in *M. tuberculosis*. TRIT was originally suggested to act as an intrinsic transcription terminator,^14^ however a recent study reported minimal overlap between TRIT sites and actual transcription termination events.^59^ Our analysis helps reconcile these findings, suggesting that this element is processed by RNase III. It is tempting to speculate that the potential involvement of RNase III in shaping transcript 3′ termini, is at least partially related to its essentiality in *M. tuberculosis*.^60^^,^^61^ Lastly, we uncovered additional 3′ terminus-associated elements, such as the Internal AUGA motif in *S. pneumoniae*. *S. pneumoniae* is unique in our data for multiple reasons: (1) its number of terminus sites exceeds the number of annotated genes (Figure 2E); (2) over 50% of its termini are Internal (Figure 2E); (3) consequently, most of its genes are associated with more than one terminus (Figure 7A); and (4) its genes are over represented in conserved condition-dependent regulation (Table S6). The AUGA motif is highly likely associated with the underlying mechanism generating these Internal termini, as it is identified in 985 sites, accounting for 61% of *S. pneumoniae* Internal termini. Although we cannot rule out a technical source to this phenomenon, an intriguing alternative is that this motif represents the recognition sequence of a hitherto unidentified toxin. Taken together, our findings underscore the rich biological information embedded in standard transcriptomic datasets and highlight the value of combining sequence data with 3′ end signals to gain mechanistic insights. As deep learning models are increasingly applied to address complex biological questions, we believe that directly integrating these two modalities holds great potential for uncovering additional layers of regulation.

The spectrum of motifs associated with the generation of 3′ termini raises intriguing evolutionary questions. One such aspect is the presence of intrinsic terminators with non-canonical stem sequences. While the landscape of possible sequences suitable to form transcription terminators is large, in five species we found a widespread use of specific sequences. In *N. meningitidis*, *N. gonorrhoeae*, *H. influenzae*, and *A. actinomycetemcomitans* the terminator sequences showed very little variation and corresponded to DNA uptake sequences (Table S5), known to serve other biological functions.^15^^,^^33^ *S. epidermidis* exhibited a different, more variable stem-associated motif (Tables S4 and S5), but still retained high information content. While we could not attribute a known function to this motif, given that the above highly preferred stem sequences had alternative biological roles, it is plausible that this sequence is also subject to other functional constraints. Another intriguing aspect concerns the evolution of transcription terminators. Conceptually, altering or replacing a terminator structure is likely to be a delicate evolutionary process, as changes in termination can affect transcript stability and the expression of the downstream gene. For this reason, our observation of diverse transcription termination mechanisms across orthologous genes (Figure 5) was surprising. This finding raises important questions about how 3′ termini and their associated termination signals evolve over time without disrupting essential regulatory functions.

The distribution of inferred transcription termination mechanisms varied markedly across species (Figure 5). Four *Streptococcus* species lacked a detectable *rho* orthologue in our data (STAR Methods), consistent with observations by Moreira et al.^62^ In these species, 9–23% of genes with a defined Primary terminus lacked intrinsic terminator features. This non-negligible fraction of unexplained termini suggests that, beyond intrinsic and Rho-dependent mechanisms, additional processes contribute to 3′ end formation. Among these, pTAMs derived from insertion sequences and/or mobile elements frequently coincided with Primary termini (Table S5), consistent with a previous suggestion for their potential role as terminators.^63^ Additional pTAMs for which we could not attribute a known mechanism, may potentially reflect weak or inefficient intrinsic terminators,^64^ Rho-dependent termination,^1^ bidirectional terminators,^19^ processing products, or other uncharacterized features. Together, these findings suggest that other mechanisms, alternative to the two major termination mechanisms may play a more substantial role in the generation of Primary termini than generally acknowledged.

In summary, our work provides a valuable platform for studying transcription termination and processing across both growth conditions and bacterial species. By making these data accessible through a user-friendly webserver (https://bacterminome.huji.ac.il), we enable to explore 3′ termini, focusing on specific genes, on particular species, or, more broadly, on the mechanisms underlying terminus generation. Our findings illustrate the power of applying unconventional analyses to widely available RNA-seq data, an approach that holds promise as deep learning methods continue to advance.

### Limitations of the study

As the major limitations of our method are important for the interpretation of our results, we explicitly addressed them in the opening paragraphs of the Discussion. In addition, the RNAtag-seq protocol, as well as many other RNA-seq protocols, is prone to PCR duplications. As the RNAtag-seq experimental protocol involves a random fragmentation step, it is more likely to include such duplications in comparison to other experimental protocols dedicated to the identification of 3′ termini. Still, we have previously shown that less than 10% of all 3′ termini and ∼2% of the primary termini detected by TRS applied to RNAtag-seq data were based on reads suspected as PCR duplications.^23^

While many of our conclusions regarding specific mechanisms and gene candidates are highly supported computationally, they would benefit from specific experimental validation. Two examples are: (1) at the mechanism level, the suggested cleavage of the TRIT element by RNase III can be tested *in vitro* or in RNase III mutant cells; (2) at the gene level, northern analysis under the relevant conditions can support putative condition-dependent termini of specific genes.^23^

## Resource availability

### Lead contact

Further information and requests for resources should be directed to and will be fulfilled by the lead contact, Hanah Margalit (hanahm@ekmd.huji.ac.il).

### Materials availability

This study did not generate new unique reagents.

### Data and code availability

• This paper analyzes existing, publicly available data, accessible at GEO: GSE15229.
• The original code has been deposited at Zenodo: https://doi.org/10.5281/zenodo.17777547 and is publicly available as of the date of publication.
• Any additional information required to reanalyze the data reported in this paper is available from the lead contact upon request.
• The atlas of bacterial 3′ termini is publicly available online at https://bacterminome.huji.ac.il.

## Acknowledgments

This study was supported by the 10.13039/501100000781European Research Council Advanced Grant #833598 (H.M.). and the 10.13039/501100003977Israel Science Foundation Grant #1253/22 (H.M.). A.B. was partially supported by a fellowship of the Data Sciences program of the Planning and Budgeting Committee of the Israel Council for Higher Education. We thank Itay Warshavsky for his assistance in mapping the sequencing datasets, and Tal Pupko and Liran Carmel for helpful discussions.

## Author contributions

Initiated and supervised the study – H.M.; methodology – H.M. and A.B.; computational analysis – A.B. and M.E.; data exploration – A.B. and Y. A.; software development – A.B.; site development – A.B.; writing – original draft – H.M., A.B., and Y.A.; review and editing – H.M., A.B., Y.A., and M.E.

## Declaration of interests

The authors declare no competing interests.

## Declaration of generative AI and AI-assisted technologies in the writing process

During the preparation of this work, the author(s) used “ChatGPT” in order to improve the readability and language of the manuscript. After using this tool, the authors reviewed and edited the content as needed and take full responsibility for the content of the published article.

## STAR★Methods

### Key resources table

**Table:** 

REAGENT or RESOURCE	SOURCE	IDENTIFIER
**Deposited data**		

Sequencing reads of the PATHOgenex database	Avican et al.^25^	GSE152295
Atlas generation and analyses code	Zenodo	https://doi.org/10.5281/zenodo.17777547

**Bacterial and virus strains**		

Bacterial strains analyzed in this study	According to Avican et al.^25^ see Table S1	N/A

**Software and algorithms**		

Fasterq-dump (version 3.0.3)	https://trace.ncbi.nlm.nih.gov/Traces/sra/sra.cgi?view=software	https://github.com/ncbi/sra-tools
bwa (version 0.7.17-r1188)	https://doi.org/10.1093/bioinformatics/btp324	https://bio-bwa.sourceforge.net/
cutadapt (version 3.5)	https://doi.org/10.14806/ej.17.1.200	https://cutadapt.readthedocs.io/en/stable/
EggNOG v5 (emapper version 2.1.12)	https://doi.org/10.1093/nar/gky1085	http://eggnog5.embl.de/#/app/home
TRS (version 1.0.0)	https://doi.org/10.1038/s41467-023-43534-2	https://github.com/amirbarHUJI/TRS
Pybedtools	https://doi.org/10.1093/bioinformatics/btr539	https://bedtools.readthedocs.io/en/latest/
Biopython (version 1.83)	https://doi.org/10.1093/bioinformatics/btp163	https://biopython.org/
MEME (version 5.5.5)	https://doi.org/10.1093/nar/gkp335	https://meme-suite.org/meme/tools/meme
pymemesuite (version 0.1.0-a3)	N/A	https://github.com/althonos/pymemesuite
MAFFT (version 7.526)	https://doi.org/10.1093/molbev/mst010	https://mafft.cbrc.jp/alignment/server/index.html
RNAfold (version 2.7.0)	https://doi.org/10.1186/1748-7188-6-26	http://rna.tbi.univie.ac.at/cgi-bin/RNAWebSuite/RNAfold.cgi
RNAalifold (version 2.7.0)	https://doi.org/10.1186/1471-2105-9-474	http://rna.tbi.univie.ac.at/cgi-bin/RNAWebSuite/RNAalifold.cgi
R2R (version 1.07)	https://doi.org/10.1186/1471-2105-12-3	https://sourceforge.net/projects/weinberg-r2r/
RNA canvas	https://doi.org/10.1093/nar/gkad302	https://rna2drawer.app/
BV-BRC (version 3.57.31)	https://doi.org/10.1093/nar/gkac1003	https://www.bv-brc.org/
FigTree (version 1.4.4)	N/A	https://tree.bio.ed.ac.uk/software/figtree/
GOATOOLS (version 1.6.5)	https://doi.org/10.1038/s41598-018-28948-z	https://github.com/tanghaibao/goatools

### Experimental model and study participant details

This study analyzed previously generated sequencing data from the PATHOgenex dataset.^25^ The bacterial strains and growth conditions are listed in Table S1. No new experiments were conducted in this study.

### Method details

#### Mapping of sequencing reads

Sequencing adapters were removed using cutadapt^65^ version 3.5 (-m 25 -q 0 -a AGATCGGAAGAGC -n 5 -e 0.15 -j 0), and processed reads were mapped to their respective reference genome (Table S2) using bwa^66^ version 0.7.17-r1188 (bwa aln -n 2 -t 8 -R 200 followed by bwa samse/sampe with ‘-a 1500 -P’ for single- or paired-end sequencing, respectively).

#### Determination of 3′ termini

The 3′ termini were determined by applying the TRS algorithm^23^ (-t 0.01 -d 70 -min_count 10 -min_height 0.1) to RNAtag-seq data (triplicate libraries) of each bacterial species and growth condition in the PATHOgenex dataset.^25^ In brief, TRS determines 3′ terminus sites by identifying characteristic peaks in RNAtag-seq data.^23^ The 3′ terminus sites were considered as such if they passed the statistical test of TRS in all three libraries of a bacterial species under a growth condition. Sites that passed the statistical test in only two libraries were also included if their “termination efficiency score” ($\bar{R_{i}}$)^23^ was ≥ 0.5. Next, for each bacterial species, we combined the 3′ termini that were determined for the different growth conditions into a single table. Since determined 3′ terminus positions may vary between growth conditions by a few nt, 3′ terminus positions that were at most 10 nt apart were merged, reporting their position as the rounded median of the positions of close termini. A stricter filtering process was applied to 3′ termini identified only in a single growth condition, retaining only 3′ termini that passed the statistical test in all the three libraries of the growth condition with “termination efficiency score” ≥ 0.6. Finally, 3′ termini assigned to tRNA and rRNA genes were omitted.

#### Determination of orthologous genes

For each of the 32 bacterial species, all annotated RefSeq genes,^67^ were uploaded to the EggNOG v5 server^27^ (emapper version 2.1.12^26^). This resulted in an assignment of a cluster of orthologous group (COG) IDs and a “Preferred_name” (when exists) for each gene in each species. Genes assigned an identical “Preferred_name” were considered orthologous genes. This generated a different set of orthologue groups compared to grouping COG IDs, and a smaller number of genes with an assigned orthologue. Nevertheless, it was preferred as a gene to “Preferred_name” is a 1:1 assignment compared to 1:N in the case of COG IDs.

#### Annotation of 3′ termini

Following Bar et al.^23^ with slight modifications, the 3 termini were assigned one of eight genomic annotations. The annotation of a 3′ terminus was based on its position relative to genes and operons (Figure S1A). The lists of genes and the gene boundaries for each bacterial species were defined according to their respective RefSeq annotation data.^67^ Note that in the downloaded RefSeq data, all protein coding genes were annotated by their CDS boundaries. Since RefSeq annotation did not include transcription unit information, genes were heuristically defined to reside within the same operon if their genomic distance was less than 100 nt. The eight genomic annotations were classified into three major categories: (1) Primary 3′ terminus – a 3′ terminus located downstream of the stop codon in a protein coding gene or the end of a non-coding gene. This category consists of four classes: (*i*) Close primary (CP) – a 3′ terminus located in the range of 5 nt upstream of the stop codon and up to 100 nt downstream of the stop codon of a protein coding gene. (*ii*) Distant primary (DP) – a 3′ terminus located up to an extended region of 200 nt for genes with no detected CP terminus. (*iii*) Alternative primary (AP) – Similar to DP, but for genes with a detected CP terminus. (*iv*) Alternative primary in transcription unit (AP in TU) – Applied only to genes that are not last in their transcription unit (replacing CP, DP, and AP). A 3′ terminus located up to 250 nt downstream of the stop codon of a protein coding gene. (2) Premature 3′ terminus – a 3′ terminus located at the (*v*) 5′ UTR of a gene (up to 100 nt upstream of the start codon of a protein coding gene) or (*vi*) internal to a coding sequence. (3) Orphan terminus – a 3′ terminus that is not associated with any annotated gene. This group consists of 3′ terminus located (*vii*) in intergenic regions (IGR) or (*viii*) antisense to annotated transcripts (AS). In practice, there can be ambiguity in the above definitions. For example, a 3′ terminus can be located within 100 nt downstream of the stop codon of a gene and within 100 nt upstream of the start codon of the consecutive gene. To explicitly define the gene associated with the terminus, we applied the following process for each terminus in our data, stopping once a condition is met (Figure S1B): (1) We calculated the distance to the end of the closest gene upstream (stop codon of a protein coding gene or the end of a non-coding gene). This was calculated using BedTools closest^68^ (-id –D a -s). (2) Based on the distance from the closest upstream gene, we first tried to assign the 3′ terminus to the Primary category classes by the following order: AP in TU, CP, and AP/DP. (3) If it was not assigned to a Primary category, we checked whether the 3′ terminus overlaps the CDS of a gene on the same strand (BedTools intersect,^68^ i.e., bedtools intersect -loj -s -wb). If it was found to reside within the CDS at a distance > 5 nt from the stop codon, it was assigned to the Premature Internal category. If it was found to reside within the last 5 nt of the CDS, it was considered a CP terminus or AP in TU based on the genomic context. (4) If the terminus was not Primary or Internal, we calculated the distance to the closest downstream gene start codon (BedTools closest,^68^ i.e., bedtools closest -iu –D a -s). If the terminus resided within 100 nt upstream of the start codon of the gene, it was assigned to the Premature 5′ UTR category. (5) If it was located in antisense to an annotated gene (BedTools intersect,^68^ i.e., bedtools intersect -loj -s -wb), it was assigned to the Orphan Antisense category. (6) Lastly, if the terminus was not assigned to any of the above, it was considered an Orphan IGR terminus.

Note that the annotation assignments depend on the selected distance parameters. In particular, we observed instances of Primary termini associated with long 3′ UTRs that are classified as Premature 3′ termini of a downstream gene or as Orphan termini. To overcome this ambiguity, we refined the process by utilizing information derived from the sequencing read data (Figures S1C and S1D). When paired-end data were available (22 out of 32 bacteria), there was additional information on the read boundaries, as the read defines both the 3′ and 5′ termini of the sequenced transcripts regardless of their length. If the 5′ end is mapped to a gene upstream of an Orphan or a Premature classified 3′ terminus, it is more likely that this 3′ terminus is actually a Primary terminus (either AP in TU, DP or AP) of the upstream gene only at a distance exceeding the original class definitions. Thus, for each Premature or Orphan 3′ terminus, we returned to the 5′ boundaries of the reads and recorded the annotation of the 3′ end, i.e., the gene it was associated with or an intergenic region. Premature termini were then re-annotated as Primary if the majority of the reads originated in an upstream gene, and Orphan termini were re-annotated as Primary if there was at least one read that originated from an upstream gene (Figure S1C). Note that we set a more stringent threshold for Premature termini compared to Orphan termini, as there is more potential of ambiguity for the former.

The above reannotation could not be applied to the ∼33% of the bacterial species, sequenced with single-end reads. Consequently, single-end and paired-end sequenced bacterial species could differ in the assignments of 3′ termini to genes. This can affect downstream analyses and can be especially important when comparing 3′ termini of orthologous genes. To reduce potential biases resulting from the difference in termini annotation, we designed a process to project the re-assignment information of 3′ termini defined by paired-end libraries to 3′ termini that are defined by single-end reads and reside on orthologous genes. Thus, for termini that were re-annotated as Primary by paired-end sequencing, we recorded the 3′ UTR length and genomic context, i.e., the immediate genes upstream and downstream of the terminus. Next, for each Premature and Orphan annotated terminus in a single-end sequenced bacterial species that is found in the same genomic context as a re-annotated Primary terminus of an orthologous gene, we re-annotated the terminus to Primary if it was within the maximal 3′ UTR length (across all orthologues in that genomic context) plus 20 nt (Figure S1D).

Note that the small number of non-coding RNAs (ncRNAs), annotated in the downloaded RefSeq data, were treated identically to coding genes. However, for ncRNA the terminology changes: start and stop codons are replaced with the start and end of the RefSeq annotated ncRNA, while 5′ and 3′ UTRs are replaced with extended 5′ and 3′ flanking regions. These extensions are required to detect potential processing of a premature transcript, as there are reported examples of ncRNA transcripts that are processed from longer precursors with 5′ and/or 3′ extensions (e.g.,^69^^,^^70^).

#### Determination of sequence enrichment profiles

A position specific scoring matrix was determined for each group of sequences aligned at the 3′ terminus position as an anchor. Each column of the matrix contains the normalized frequency of the four nucleotides. The normalized frequency is the observed frequency divided by the genomic nucleotide frequency in the respective bacterial species, where chromosome and plasmids (if exist) are considered. The log_2_ of the normalized frequencies was considered as the enrichment value. To obtain global nucleotide enrichment profiles across all the bacterial species, a per position weighted average was calculated. The weights of the average were calculated using the proportion of the number of sequences attributed to each species. To validate that the generated profiles are not dominated by the contributions of specific species, the analyses were repeated with regular average. The resulting highly similar profiles indicated that there was no specific species bias (Figure S2).

#### Identification of sequence motifs in the 3′ UTR

To determine sequence motifs in the 3′ UTRs of bacterial species, we first extracted for each gene with an assigned Primary terminus its 3′ UTR sequence, provided the sequence length > 35 nt. Note that for genes with multiple Primary termini, the sequence was determined by the most downstream terminus. These 3′ UTR sequences, extended by five nt downstream of the 3′ terminus, were used as input for the sequence motif discovery tool MEME.^71^ MEME background distribution was based on the input sequences (default). MEME was run with the following parameters: ‘-mod zoops -minw 6 -maxw 15 -evt 100 -rna’. For two species (*E. faecalis* and *L. monocytogenes*), which had a motif with >1000 identified sites (the default value), we added the parameter ‘-csites 2000’. Note that by limiting the motif length to 15 nt, longer motifs might be represented by multiple consecutive short motifs. The application of MEME to the entire 3′ UTR is expected to identify termination-related signals as well as signals of other, unrelated, regulatory processes. Note that ncRNAs were also included only the downstream region is not considered a 3′ UTR but a flanking region.

#### Clustering of motifs in the 3′ UTR

We applied RSAT^72^ to cluster the MEME^71^ identified motifs in the 3′ UTR of all species. By default, RSAT clustered the motifs in a strand-independent manner, i.e., forward and reverse complement motifs were clustered together. For downstream analyses such mixed clusters were divided into two clusters, each with the original orientation of the motifs.

#### Distance of MEME detected 3′ UTR motifs from Primary termini

For each motif we measured the minimal distance between the motif boundaries and the 3′ terminus for all genes it was detected in, and constructed a distance distribution. When multiple Primary termini are associated with the gene 3′ UTR, the absolute distance to the closest terminus is measured. Note that the closest terminus can be either upstream or downstream of the motif. To avoid the effect of outlier distances we characterized the distance distribution by its median rather than average. Accordingly, to assess the dispersion of the distances, we used the median absolute distance (MAD), which is more robust to outliers in the data, compared to the variance. Similarly, we characterized the distribution of the distances from the stop codon to the motif by its median and MAD values.

Motifs with a median distance ≤15 nt from the 3′ terminus were defined as primary Termini Associated Motifs (pTAMs). MAD was used for the manual association of detected motifs to previously reported elements (Table S5).

#### Ter-peak score along the motif region

For each reported MEME^71^ motif, we retrieved the sequences comprising the motif of length ‘W’ with ‘M’ flanking nt on both sides (for Primary motifs M=20, for Internal motifs M=25). The number of the sequences containing the motif is denoted by ‘N’. These sequences were aligned, anchored at the motif start position. To calculate the ter-peak score, we returned to the raw read start count data, which correspond to 3′ terminus signals. We extracted for each of the bacterial species’ libraries (‘l’), the number of read start counts at each position (‘i’) of each aligned sequence (‘s’) and denote it *X*_*s*,*l*,*i*_. Then a two-step normalization was applied: First for each library, we divided the per position read start count (*X*_*s*,*l*,*i*_) by the total read start count in the respective aligned sequence ($\sum_{i=1}^{W+2\cdot M}X_{s,l,i}$), and generated per position relative frequencies *f*_*s*,*l*,*i*_. Note that libraries with a total read start count < 50 were omitted from the analysis. Second, for each position of the aligned sequence, the relative frequencies were averaged over all the relevant libraries (‘L’) (${\bar{f}}_{s,i}=\frac{1}{\left|L\right|}\cdot \sum_{l\in L}f_{s,l,i}$). This resulted in a normalized read start distribution per position ‘i’: ${\bar{f}}_{1,i},{\bar{f}}_{2,i},\ldots ,{\bar{f}}_{N,i}$. Finally, the median of each positional distributions was the ter-peak score of this position.

For three sequence elements, TRIT in *M. tuberculosis* and the CR and DNA uptake sequence containing terminators in the *Neisseria* species, we extended the ter-peak calculation from a motif scope to the reported element scope. The extension of the ter-peak calculation was as follows: for the TRIT we extracted the sequence spanning five nt upstream of the motif matching the element start and 45 nt downstream, covering the entire element region. The CR element boundaries were defined by constant two enclosing elements that could vary in their distance up to ∼160 nt in total.^73^ Thus, we extracted the sequence spanning the detected motif matching the CR start sequence to 200 nucleotides downstream of the motif. Since this region may exceed the element boundaries, depending on the variable region length, we looked for the terminal part of the enclosing element sequence 5′-CTATA-3′^73^ and trimmed the sequence accordingly. One sequence out of 69 did not contain the second enclosing element and was omitted from the analysis. In most occurrences of the DNA uptake sequence motif, it statistically significantly co-occurred with a reverse complement motif (*p*-value of 3.31E-143 by a hypergeometric test). Thus, for the DNA uptake sequence terminator element, we extracted the sequence beginning at the DNA uptake sequence motif and five nt downstream its reverse complement motif. The multiple sequence alignment for each of the three elements was obtained using MAFFT^74^ (with the -auto flag; version 7.526). Aligned sequences with unique insertions (i.e., insertions that were not found in 90% of the aligned sequences) were excluded. This resulted in the removal of 3 out of 75 sequences in *M. tuberculosis* TRIT element, 55 out of 471 sequences in *N. gonorrhoeae* DNA uptake sequence, and 1 out of 32 and 1 out of 68 in *N. gonorrhoeae* and *N. meningitidis* CR element, respectively. Using the remaining sequences, the multiple sequence alignment was performed again. Note that these alignments contain gaps and thus the ter-peak calculation had to be adjusted. Ter-peak is calculated as previously, except for the median that is taken over the scores of all non-gapped positions.

In addition, we defined a 3′ UTR ter-peak score for individual genes. While in the ter-peak calculation we compute the positional read start frequency of individual motif site ${\bar{f}}_{s,i}$ over the region of the motif with flanking nucleotides, here we use the entire gene 3′ UTR. The positional score is simply ${\bar{f}}_{s,i}$ and not a positional median, as we consider only one sequence.

In parallel, the alignments were used to study the nucleotide distribution at each position (presented by Logo representation). Note that for the Logo representation, gapped positions of the TRIT, CR and DNA uptake sequence motifs were treated as non-gapped positions, where only A/C/G/U were considered and their relative frequencies were in respect to all aligned sequences.

#### Testing for co-occurrence of motifs

Co-occurrence of motifs was calculated per species. To test whether two sequence motifs appear with one another (regardless of genomic order) more than expected at random, we applied a Hypergeometric test in which N is the total number of genes analyzed by MEME,^71^ M is the number of genes with the first motif, L is the number of genes with the second motif, and K is the number of genes with both sequence motifs. The applied test is one sided and corrected for multiple hypothesis testing using Bonferroni correction.

#### Generation of consensus structures of TRIT and CR elements

To generate the consensus structure of the TRIT (Figure 3D) and CR (Figure S5C) motifs, we used RNAalifold^75^ with the multiple sequence alignment we produced to create their ter-peak profile. The consensus structure output of RNAalifold and the multiple sequence alignment were then used as input to R2R^76^ to generate the structure figures.

#### Estimation of intrinsic termination by the U-tract length

Dar and Sorek (2018)^4^ generated an experimental dataset of Rho-dependent and independent (intrinsic) gene terminators in *E. coli* BW25113 and showed that the U-tract length is a major feature distinguishing between the two terminator classes. For each terminus reported in Dar and Sorek dataset, we calculated the U-tract length in the sequence in vicinity of the reported 3′ terminus sites (as described below). We found that intrinsic terminators tended to have a U-tract of at least 5 Us and set this threshold as an approximation for intrinsic termination (Figure 5A).

#### Extraction of U-tract length

For each gene, we analyzed its Primary termini, considering each Primary terminus located at least 20 nt from the boundary of its respective gene. For each such terminus, we searched within a a region of 25 nt upstream and 10 nt downstream of the terminus site the longest U-tract that fulfills two conditions: (1) the U-tract may begin at most 2 nt downstream of the determined position; (2) the U-tract may end at most 5 nt upstream of the determined position. The longest U-tract was defined as the maximal number of consecutive uridines with at most a single nucleotide gap (the gap is not counted). Thus, a U-tract with the sequence UUUAU was considered a U-tract of length 4, and UUUAUAU is considered a U-tract of length 4. If there are multiple Primary termini for the same gene, the U-tract with the maximal length is selected.

#### Classification of pTAMs by sequence and structure elements

pTAMs were classified in a hierarchical manner. First, pTAMs containing a U-tract of ≥5 nt within their sequence were designated canonical intrinsic terminators. If no such tract was present within the pTAM itself but appeared in the downstream flanking sequence, it was classified as a non-canonical intrinsic terminator. pTAMs matching either the CR element or TRIT consensus were designated putative cleavage substrates. Remaining pTAMs were assigned to the unclassified category. Following close examination, three unclassified pTAMs and two U-motif pTAMs were manually reclassified as non-canonical intrinsic (Table S4).

#### Classification of genes by pTAMs and termination mechanisms

Genes were classified by their pTAMs and termination mechanisms using a hierarchical approach. Note that while pTAMs were defined by their median distance from the 3′ terminus, the sites of individual genes could deviate from this distance. Thus, each individual site was re-evaluated and only pTAM sites with a distance≤15 were considered. We prioritized pTAM types in the following order: U-motif, non-canonical intrinsic, putative cleavage substrates of RNase III, and unclassified mechanism. Next, if no pTAM could be assigned, the termination mechanism was inferred from the U-tract length (>4 nt for intrinsic termination, otherwise alternative mechanism).

#### 3′ UTR structure prediction and visualization

To predict 3′ UTR structures, we applied RNAfold 2.7.0^77^ with default settings to the 3′ UTR sequence with flanking 3 nt downstream. The output structure and the 3′ UTR sequence were used as input to RNAcanvas^78^ and the nucleotides were colored by the ter-peak values calculated for this region.

#### Construction of a phylogenetic tree

The phylogenetic tree (Figure S7A) was constructed using the bacterial and viral bioinformatic resource center (BV-BRC)^79^ bacterial phylogenetic tree service with default parameters. Since we utilize only phylogenetic distances in our analyses and do not rely on the tree topology, the tree was unrooted and visualized using FigTree v1.4.4.

#### Exponential decay fit

To fit the regression line for the relationship between the termination profiles similarity scores and the phylogenetic distance, we used the exponential decay function f(x)=a·*e*^−*b*·*x*^+c. The line was fitted using python’s curve_fit function with default parameters by providing all pairs of species regardless of the statistical significance of the similarity score.

#### Identification of sequence motifs around internal termini

While Primary termini naturally define a search region for motifs associated with 3′ termini, i.e., the 3′ UTR, it is not the case for Internal termini. Thus, to determine sequence motifs around Internal termini of bacterial species, we extracted the 25 nt flanking each side of the terminus. When multiple internal termini resided within this distance, we took an extended sequence spanning the most upstream to the most downstream positions of these regions. Similar to the sequence motif identification in the 3′ UTR, we used MEME^71^ to discover sequence motifs. We used the same parameters as in the 3′ UTR analysis except for the background sequence model. For the Internal termini, we randomly sampled 1,000 non overlapping sequences from the CDS in the genome and used a second order Hidden Markov Model generated using the ‘fasta-get-markov’ utility of the MEME-suite.

The run of *S. pneumoniae* differed from the others, as we used the ‘anr’ model instead of ‘zoops’, enabling the identification of multiple occurrences in the same sequence. This was done as we observed that the internal motif in this species is short and sometimes occurs multiple times.

#### Filtering internal terminus associated motifs (iTAMs)

In contrast to pTAMs which were defined by their distance from the 3′ terminus, Internal motifs are, by definition, close to Internal termini. Therefore, iTAMs are not defined by their distance from the terminus, rather we used their ter-peak score. Specifically, we calculate the ter-peak score in a region including the motif sequence with ±25 nt flank. The maximal signal in the region was used to calculate the log_2_ fold change with respect to a uniform distribution. Note that the uniform distribution depends on the motif size as it dictates the region length. Motifs with a log_2_ fold change ≥ 2, were considered iTAMs. Since multiple site detection per sequence was allowed in the MEME^71^ run of *S. pneumoniae*, we excluded sequences with multiple sites to avoid ambiguities of the ter-peak analysis.

#### Identification of orthologous genes with conserved condition-dependent termini

We focused on condition-dependent changes between Premature and Primary termini. For this analysis, we excluded Premature termini that are located closer to the stop codon than to the start codon of the gene. To quantify the relative use of the Premature terminus compared to the Primary terminus between growth conditions, we used the cumulative distribution function (CDF) of read starts that represent 3′ ends of fragments. i.e., in each library, we calculated the fraction of the number of all read starts upstream of and including the premature terminus out of the total number of read starts in the region. The region used to calculate the CDF spans either the start codon or 100 nt upstream of the Premature terminus (the further upstream position of the two) to the Primary terminus. For this analysis, we excluded all libraries with read count in the region below a minimal threshold of 100 reads, and then analyzed only growth conditions with at least two libraries. The growth condition score was the average CDF value at the Premature terminus across its libraries. The between-condition score is the absolute difference between the scores of the two compared growth conditions. Pairs of orthologous genes with between-condition scores of ≥0.3 in each respective species were considered candidates for having conserved condition-dependent termini.

#### GO term enrichment of conserved condition-dependent genes

To test for enrichment of GO terms in the genes with conserved condition-dependent termini, we extracted the set of GO terms associated with each group of orthologous genes in our data using EggNOG emapper (version 2.1.12^26^). For this analysis, we limited the total population to the set of genes with an orthologue in at least one other species. Enrichment calculations were done using the GOATOOLS python package^80^ using FDR correction for multiple hypotheses.^28^ GO slims prokaryote dataset^81^^,^^82^ (version dated 2026-05-19) was used as input to the GOATOOLS to report higher-level annotations.

### Quantification and statistical analysis

The statistical details of individual analyses are provided in the text or in the corresponding figure legends, tables, and method details sections. Where applicable, these details include the statistical test, sidedness, *p*-value or adjusted *p*-value and multiple-testing correction method.

## Supplemental information

Document S1. Figures S1–S7 and Table S5

Table S1. Bacterial species and growth conditions used in this study, related to Figure 1A

Table S2. Number of reads in the sequencing libraries

Table S3. The atlas of 3′ termini

Table S4. Motifs detected in the 3′ UTR of bacterial species

Table S6. Internal motifs detected in bacterial species

Table S7. Candidates of conserved condition-dependent regulation by alternative 3′ termini

Document S2. Article plus supplemental information
