# Supplementary material for: Cross-species atlas of bacterial RNA 3′ ends reveals termination mechanistic signatures and evolutionary patterns
Source: Cell Rep. 2026 Oct 17;45(10):118076. doi: 10.1016/j.celrep.2026.118076 (PMC13714241; doi:10.1016/j.celrep.2026.118076)
Supplement: Supplementary file 1 [file mmc1.pdf]

Cell Reports, Volume *45*

Supplemental information

Cross-species atlas of bacterial RNA 3′ ends reveals termination mechanistic signatures and evolutionary patterns

Amir Bar, Yael Altuvia, Michal Eldar, and Hanah Margalit

Supplementary Figures

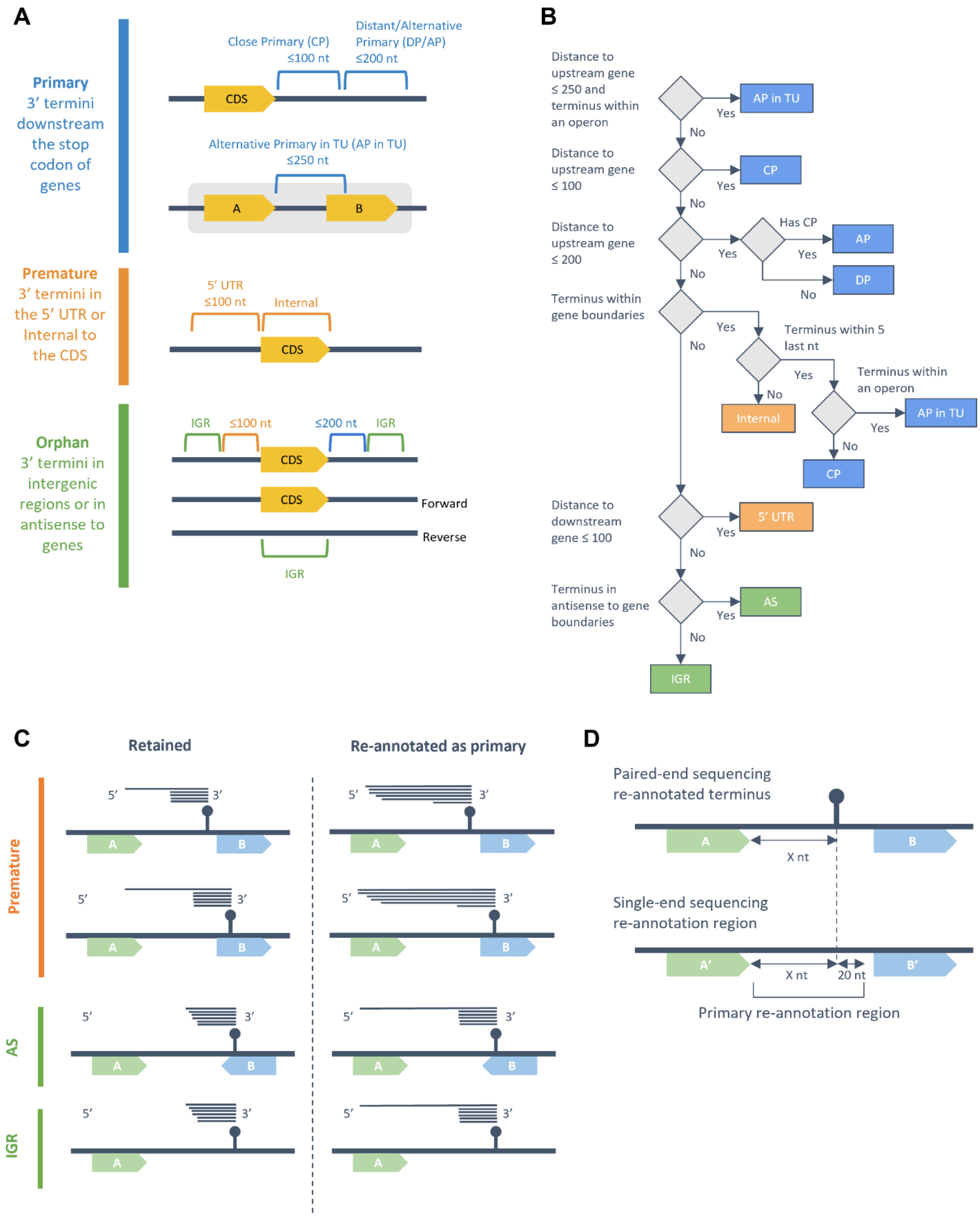

Figure S1. Annotation of 3′ termini. Related to figures 1, 2. (A) Each 3′ terminus was classified into one of three categories based on its distance from annotated genes: (1) Primary – located downstream of the stop codon of genes. Primary termini are further categorized into subcategories based on their distance from the stop codon: When a terminus is located downstream of the stop codon of the last gene in a transcription unit (TU), it will be considered Close Primary (CP) if it is at most 100 nucleotides (nt) downstream of the stop codon, or Distant Primary (DP) if it is at most 200 nt downstream of the stop codon. If a CP terminus was already assigned to a gene, a downstream DP terminus will be considered as Alternative Primary (AP) terminus. When a terminus is located downstream of the stop codon of a gene that is not last in its TU, it will be considered as Alternative Primary in TU (AP in TU) for any distance downstream of the stop codon, provided that it does not exceed 250 nt. (2) Premature – located either in the 5′ UTR of a TU, i.e., at most 100 nt upstream of the start codon of the first gene in the TU, or Internal to the coding sequence (CDS) of a gene. (3) Orphan – located in an intergenic region (IGR) or Antisense to the CDS of a gene. Note that ncRNAs were treated identically to coding genes albeit with a different terminology. Start and stop codons are replaced with the gene start and end of the RefSeq [2] annotated ncRNAs, while 5′ and 3′ UTRs are replaced with extended 5′ and 3′ flanking regions (STAR methods). (B) Flowchart outlining the 3′ terminus annotation algorithm and its prioritization criteria when annotations are ambiguous. (C) Illustration of the decision making in the process of 3′ terminus re-annotation by paired-end sequencing data. Retained annotation (left panel), revised annotation (right panel). Premature termini of gene B are re-annotated to Primary of gene A, if the majority of reads extend to gene A. Antisense and IGR termini are re-annotated to Primary by any evidence of reads that extend to gene A (i.e., even a single extended read is a sufficient evidence). Overall, 1,062 Premature and 4,583 Orphan 3′ termini were re-annotated as Primary, comprising ~12% of 45,696 3′ termini initially classified as either Premature or Orphan in species with paired-end sequenced libraries. (D) Re-annotation of Premature and Orphan 3′ termini in single-end sequenced bacterial species based on the re-annotation of 3′ termini in paired-end sequenced bacterial species. A terminus that was re-annotated to an upstream gene (gene A) by the paired-end sequencing reads, is considered for re-annotation in single-end sequenced species if the genomic context is retained (the immediate upstream and downstream genes flanking the terminus are orthologous between the species). For these candidates, we denoted by ‘x’ the distance between

the terminus and the upstream stop codon in the paired-end sequenced species. We re-annotated the terminus in the single-end sequenced species if the terminus was at most x + 20 nt from the stop codon of the upstream gene. Overall, 138 Premature and 385 Orphan termini were re-annotated as Primary, comprising ~5% of 9,984 3′ termini initially classified as either Premature or Orphan in species with single-end sequenced libraries.

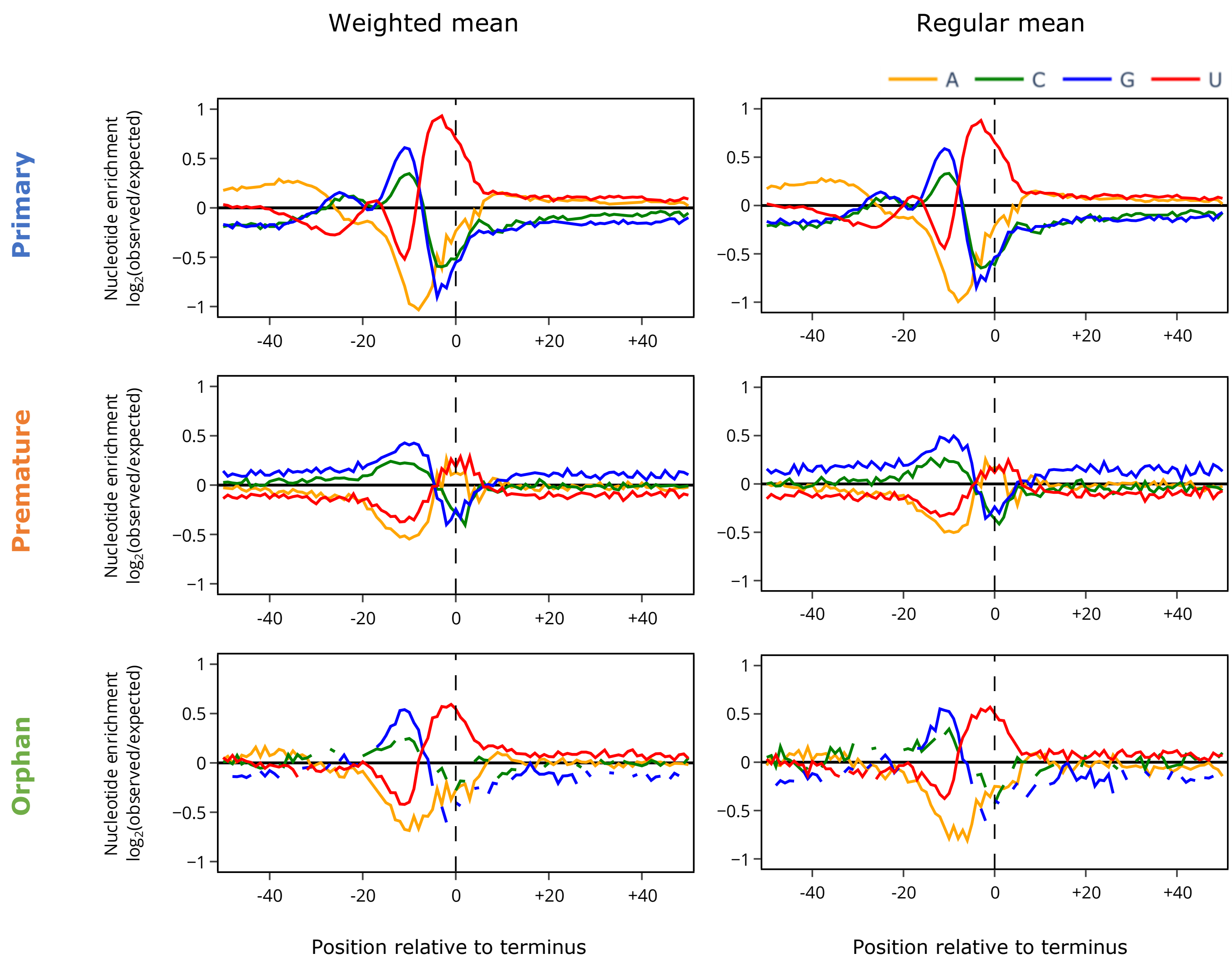

Figure S2. Comparison of methodologies for computing the global sequence enrichment profiles. Related to Figure 2C-D. The global nucleotide enrichment profiles of the bacterial species in the vicinity of 3′ termini were averaged by two methodologies – a weighted average that considers the number of termini in each species in the calculation (left panel), or a regular average (right panel), which weights equally all bacterial species. Application of the two methodologies resulted in similar nucleotide enrichment patterns, indicating that the observed pattern is not biased by the number of termini identified in a specific species.

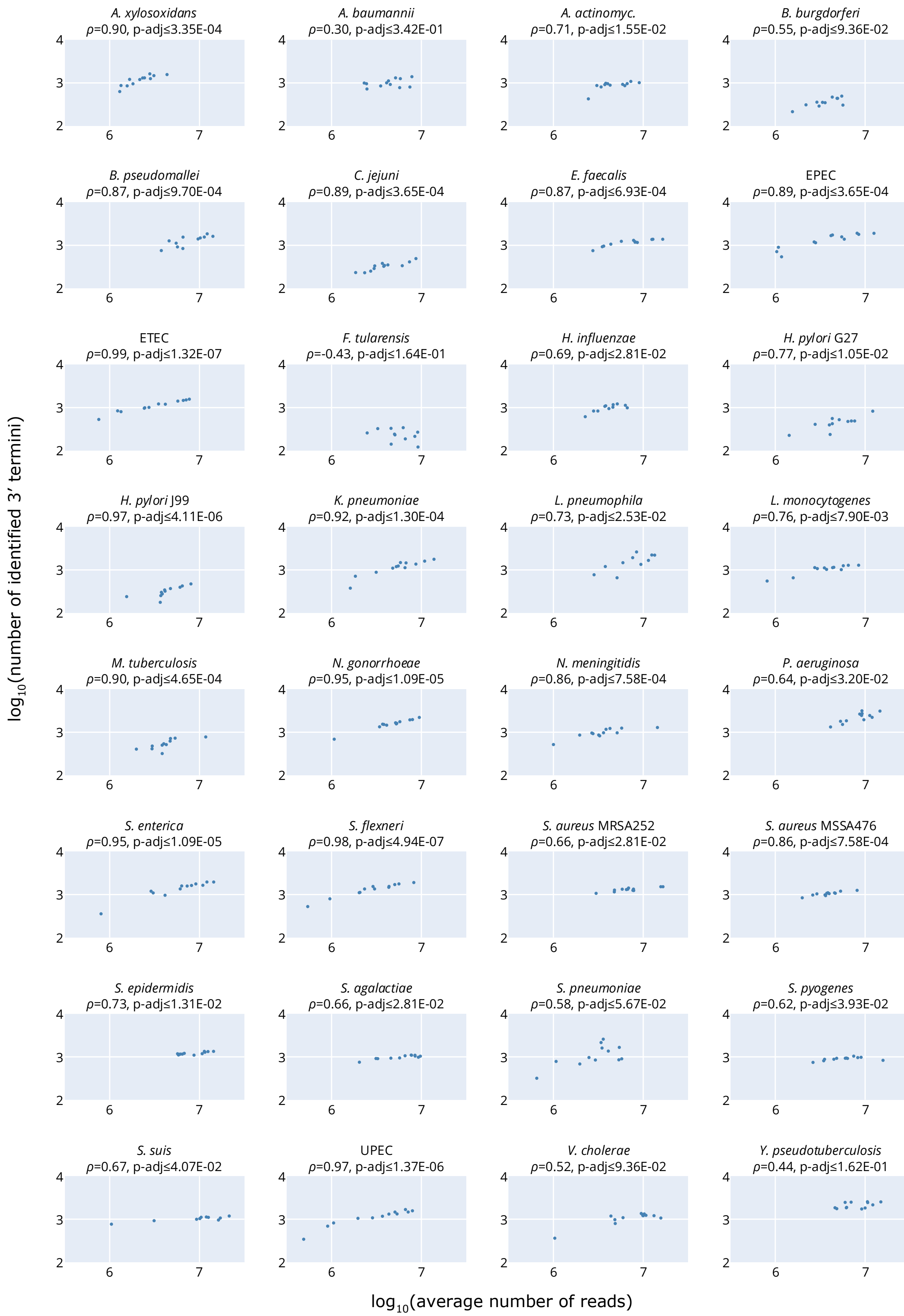
A. xylosoxidans
ρ=0.90, p-adj≤3.35E-04
A. baumannii
ρ=0.30, p-adj≤3.42E-01
A. actinomyc.
ρ=0.71, p-adj≤1.55E-02
B. burgdorferi
ρ=0.55, p-adj≤9.36E-02
B. pseudomallei
ρ=0.87, p-adj≤9.70E-04
C. jejuni
ρ=0.89, p-adj≤3.65E-04
E. faecalis
ρ=0.87, p-adj≤6.93E-04
EPEC
ρ=0.89, p-adj≤3.65E-04
ETEC
ρ=0.99, p-adj≤1.32E-07
F. tularensis
ρ=-0.43, p-adj≤1.64E-01
H. influenzae
ρ=0.69, p-adj≤2.81E-02
H. pylori G27
ρ=0.77, p-adj≤1.05E-02
H. pylori J99
ρ=0.97, p-adj≤4.11E-06
K. pneumoniae
ρ=0.92, p-adj≤1.30E-04
L. pneumophila
ρ=0.73, p-adj≤2.53E-02
L. monocytogenes
ρ=0.76, p-adj≤7.90E-03
M. tuberculosis
ρ=0.90, p-adj≤4.65E-04
N. gonorrhoeae
ρ=0.95, p-adj≤1.09E-05
N. meningitidis
ρ=0.86, p-adj≤7.58E-04
P. aeruginosa
ρ=0.64, p-adj≤3.20E-02
S. enterica
ρ=0.95, p-adj≤1.09E-05
S. flexneri
ρ=0.98, p-adj≤4.94E-07
S. aureus MRSA252
ρ=0.66, p-adj≤2.81E-02
S. aureus MSSA476
ρ=0.86, p-adj≤7.58E-04
S. epidermidis
ρ=0.73, p-adj≤1.31E-02
S. agalactiae
ρ=0.66, p-adj≤2.81E-02
S. pneumoniae
ρ=0.58, p-adj≤5.67E-02
S. pyogenes
ρ=0.62, p-adj≤3.93E-02
S. suis
ρ=0.67, p-adj≤4.07E-02
UPEC
ρ=0.97, p-adj≤1.37E-06
V. cholerae
ρ=0.52, p-adj≤9.36E-02
Y. pseudotuberculosis
ρ=0.44, p-adj≤1.62E-01
log10(number of identified 3′ termini)
log10(average number of reads)

Figure S3. The correlation between library size and identified 3′ termini. Related to Figure 2E. For each bacterial species and growth condition we determined 3′ termini by applying TRS to the respective RNAtag-seq libraries (triplicates). For each bacterial species shown is a scatter plot in which each dot represents a growth condition. The x-axis is the $\log_{10}$ average number of reads over the respective libraries. The y-axis is the $\log_{10}$ average number of 3′ termini identified for this condition. For most bacterial species (26 out of 32), there is a statistically significant correlation between the two measures. Spearman correlation coefficients (ρ) and False Discovery Rate (FDR) [3] adjusted p-values are shown for each species.

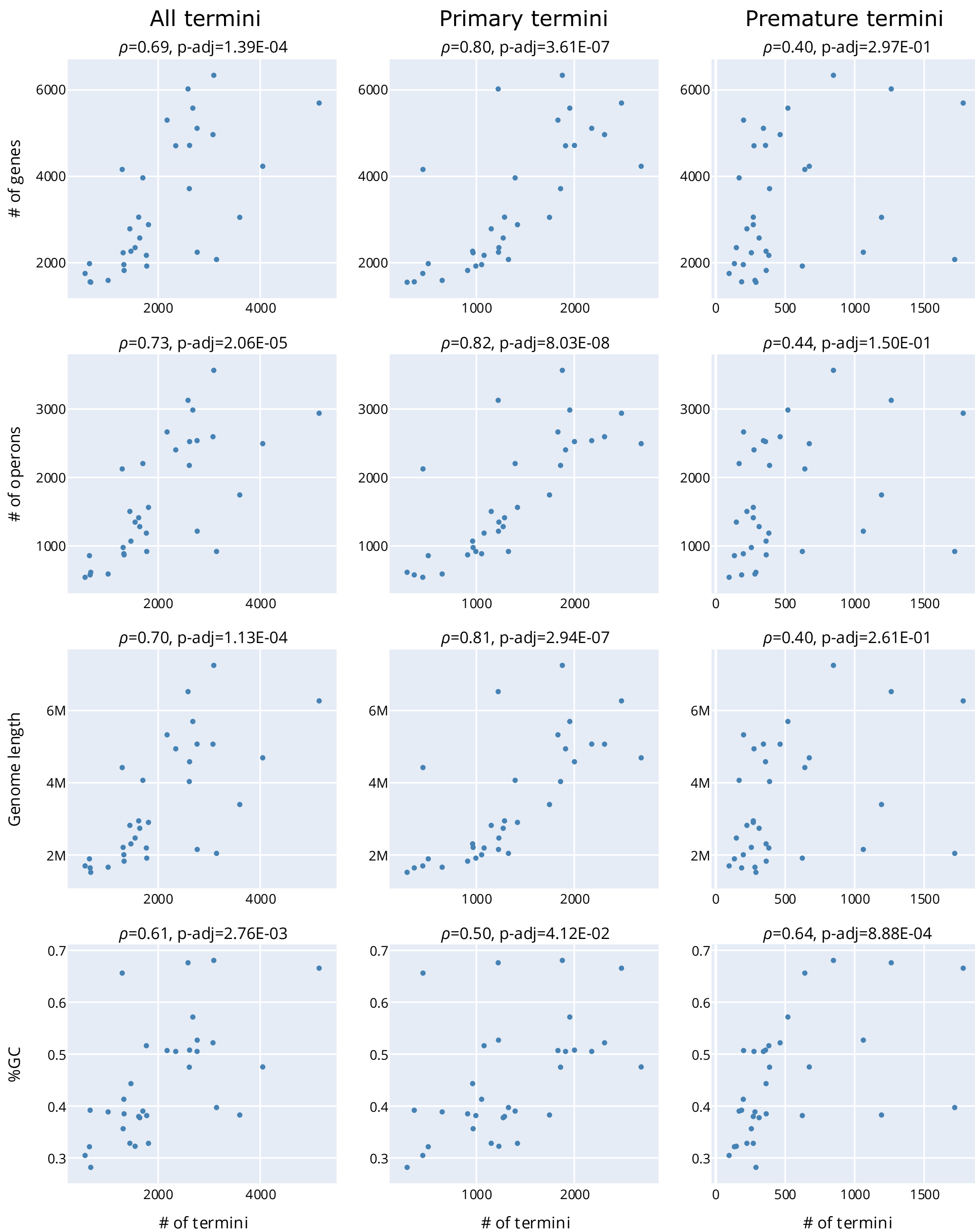
All termini
Primary termini
Premature termini
ρ=0.69, p-adj=1.39E-04
ρ=0.80, p-adj=3.61E-07
ρ=0.40, p-adj=2.97E-01
ρ=0.73, p-adj=2.06E-05
ρ=0.82, p-adj=8.03E-08
ρ=0.44, p-adj=1.50E-01
ρ=0.70, p-adj=1.13E-04
ρ=0.81, p-adj=2.94E-07
ρ=0.40, p-adj=2.61E-01
ρ=0.61, p-adj=2.76E-03
ρ=0.50, p-adj=4.12E-02
ρ=0.64, p-adj=8.88E-04
# of genes
# of operons
Genome length
%GC
# of termini
# of termini
# of termini

Figure S4. Features associated with the number of 3′ termini detected. Related to Figure 2E. To assess the effect of other genomic features on the number of 3′ termini identified in each bacterial species, we extracted for each species the number of genes and operons, the genome length, and the GC content (y-axis). These features were compared with the overall number of termini, Primary termini, and Premature termini (x-axes). Each scatter plot represents the relationship between a feature to a subset of detected 3′ termini and quantified by Spearman's correlation coefficient (*ρ*) and the adjusted p-value (p-adj; by Bonferroni correction).

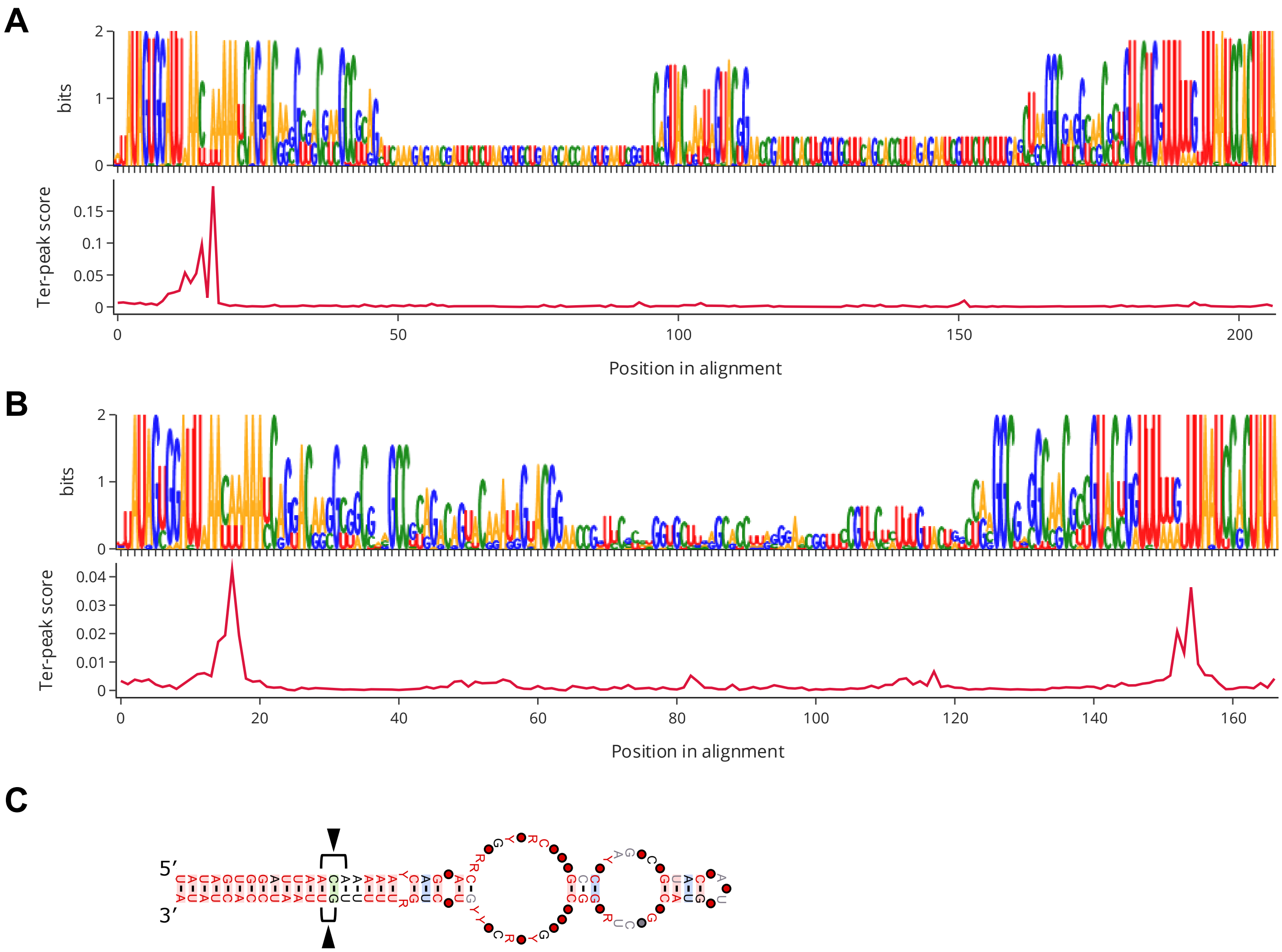

Figure S5. Sequence motifs associated with 3′ termini. Related to Figure 3. (A-B) Logo format (upper panel) and positional ter-peak scores (lower panel) of the CR element detected in the 3′ UTR of *N. meningitidis* (A) and *N. gonorrhoeae* (B). (C) The predicted structure of the CR element in *N. gonorrhoeae*. The structure is based on the multiple sequence alignment of the MEME [1] reported motif sites (STAR methods). Note that the structure is of a sequence shorter than the alignment shown in (B) since positions containing gaps in many of the aligned sequences were omitted. Arrows over brackets point to the nucleotide regions with high ter-peak scores.

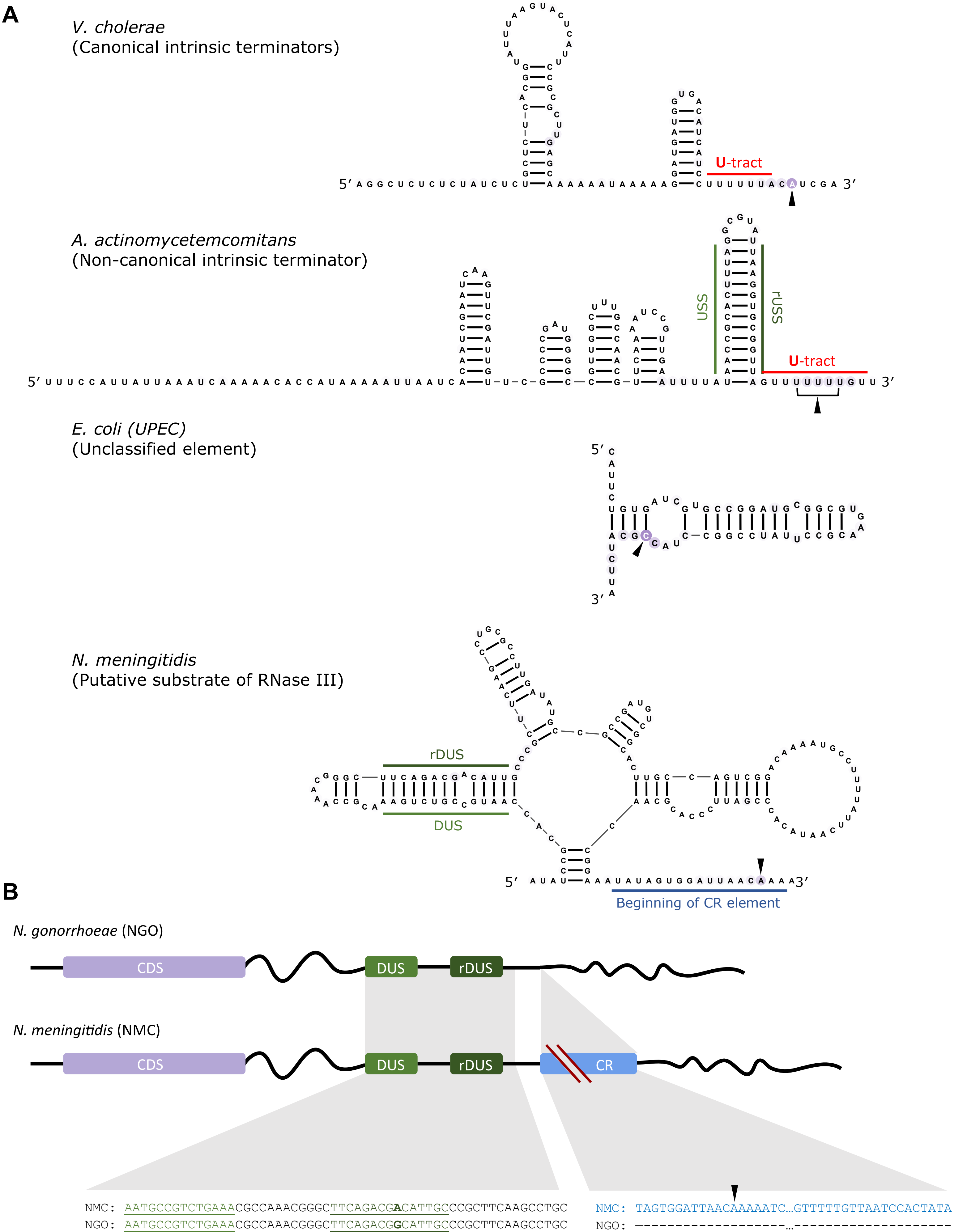
A
V. cholerae
(Canonical intrinsic terminators)
U-tract
5′ AGGCUCUCUCUAUCUCU—AAAAAAUAAAAAG—CUUUUUUACAUCGA 3′
A. actinomycetemcomitans
(Non-canonical intrinsic terminator)
USS
rUSS
U-tract
5′ UUUCCAUUAUUAAAUCAAAAACACCAUAAAAAUUAAUCA 3′
E. coli (UPEC)
(Unclassified element)
5′
3′
N. meningitidis
(Putative substrate of RNase III)
rDUS
DUS
5′ AUAU—AAAUAUAGUGGAUUAACAAAA 3′
Beginning of CR element
B
N. gonorrhoeae (NGO)
CDS
DUS
rDUS
N. meningitidis (NMC)
CDS
DUS
rDUS
CR
NMC: AATGCCGTCTGAAACGCCAAACGGGCTTCAGACGACATTGCCCGCTTCAAGCCTGC
NGO: AATGCCGTCTGAAACGCCAAACGGGCTTCAGACGGCATTGCCCGCTTCAAGCCTGC
NMC: TAGTGGATTAACAAAAATC…GTTTTTGTTAATCCACTATA
NGO: -------------------…---------------------

Figure S6. Examples of distinct elements shaping the 3′ UTR of orthologous genes. Related to Figure 4. (A) Examples of *cysE* orthologues in which we found variations in the 3′ elements shaping the 3′ UTR in different species. Shown are the predicted structures of the 3′ UTR in the different species (STAR methods). The specific elements are noted by name and are marked over the sequence. USS – uptake signal sequence; rUSS – its reverse complement; DUS – DNA uptake sequence; rDUS – its reverse complement; CR – Corriea repeat. Purple shading indicates the positional 3′ UTR ter-peak scores, with darker shading corresponding to a higher score. Arrows point to the nucleotide with the highest 3′ UTR ter-peak score, marking the implied 3′ terminus of the transcript. In the U-tract of *cysE* in *A. actinomycetemcomitans*, multiple positions showed an identical terminus signal (marked by an arrow pointing at rectangular bracket). (B) Illustrative comparison of the 3′ UTRs of *cysE* in *N. meningitidis* (NMC) and *N. gonorrhoeae* (NGO). The sequences downstream of the coding sequence (CDS) of *cysE* in both species are highly similar except for the insertion of the CR element in *N. meningitidis*. Consistently, a 3′ terminus at the middle of the CR element (marked by a black arrow on the sequence zoom-in and red breaks on the element illustration), was observed only in *N. meningitidis*.

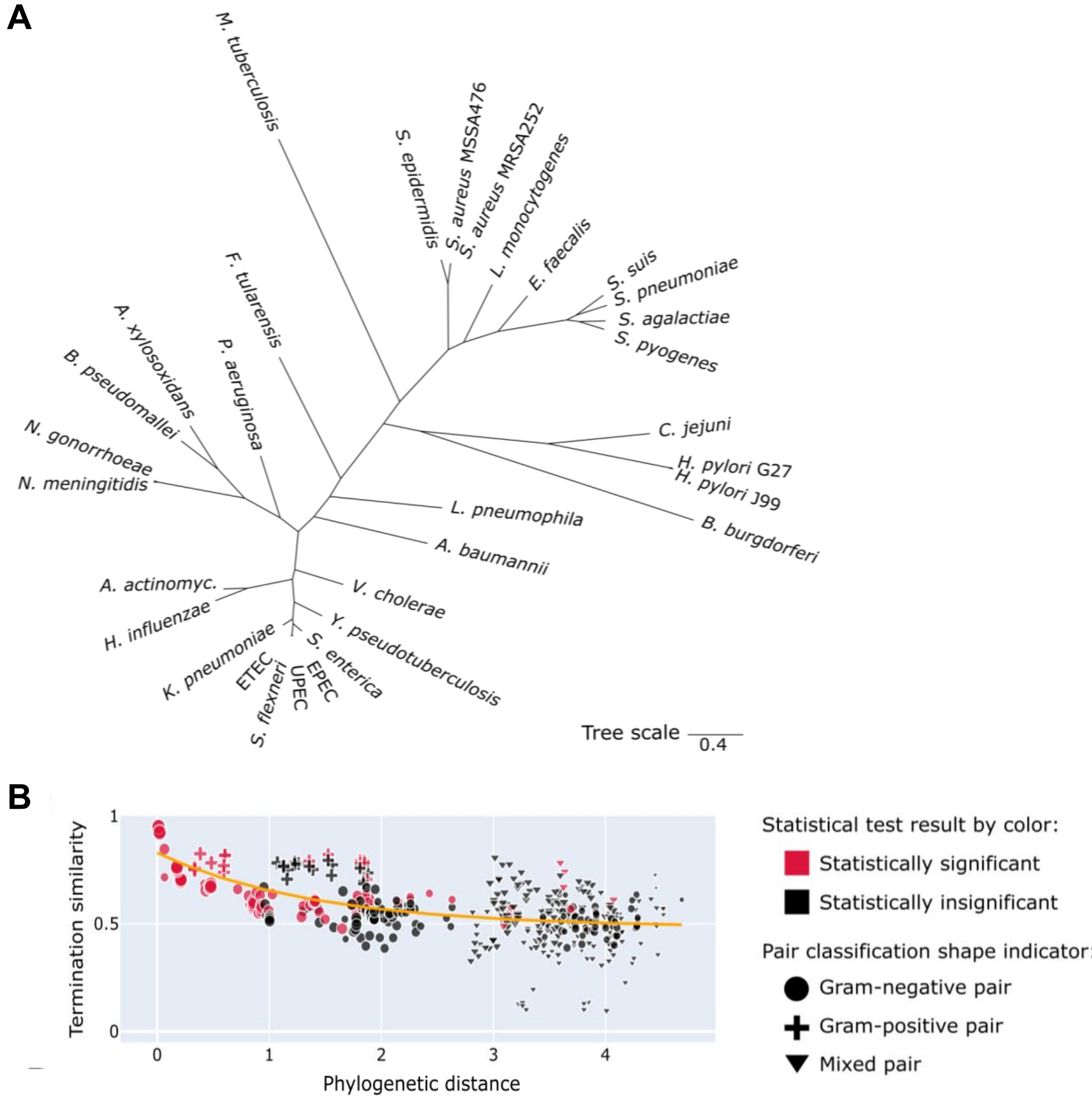

Figure S7. Conservation of the implied termination mechanism across orthologous genes. Related to Figure 5. (A) The phylogenetic tree inferred for the 32 bacterial species (STAR methods). (B) Scatter plot of the simple matching similarity score (i.e., the fraction of positions with identical values), as a function of the phylogenetic distance. Species pairs are marked as follows: plus (both are Gram-positive), circle (both are Gram-negative), and triangle otherwise (mixed pairs or both diderm). Symbol size reflects the number of orthologous genes with classified termination in both species. The statistical significance of a similarity score was assessed by applying a one-sided permutation test with 100,000 shuffles. Scores were considered statistically significant if their p-value was below 0.05 after FDR correction [3]. Symbols are colored red if the similarity score passed the statistical test and black otherwise. Orange lines indicate the regression fit of an exponential decay model (STAR Methods).

Table S5. Previously reported sequence elements matching MEME [1] detected motifs

Element	Species	References‡	Element category§
DNA uptake sequence*	*H. influenzae*	[4,5]	Non-canonical intrinsic
DNA uptake sequence*	*A. actinomycetemcomitans*	[6,7]	Non-canonical intrinsic
DNA uptake sequence*	*N. gonorrhoeae*,<br>*N. meningitidis*	[8-10]	Non-canonical intrinsic
CR/CREE/NEMIS†<br>CR – Correia Repeat<br>CREE – Correia Repeat Enclosed Element<br>Nemis – Neisseria miniature insertion sequences	*N. gonorrhoeae*,<br>*N. meningitidis*	[11-15]	Putative substrates of RNase III cleavage
SPRITE (Streptococcus pneumoniae Rho-Independent Terminator-like Element)	*S. pneumoniae*	[16]	NT-FD
BOX	*S. pneumoniae*	[17]	NT
YPAL (Yersinia palindromic elements)	*Y. pseudotuberculosis*	[18,19]	Unclassified element
STAR (Staphylococcus aureus repeat)	*S. aureus*	[20]	NT
VCR – A *V. cholerae* repetitive DNA sequence	*V. cholerae*	[21] and references therein in Figure 3A	Unclassified element
TRIT (Tuberculosis Rho-independent terminator)	*M. tuberculosis*	[22]	Putative substrates of RNase III cleavage
EFAR (Enterococcus faecalis repeat)	*E. faecalis*	[23-25]	Non-canonical intrinsic
Issep1 (Family IS1182)	*S. epidermidis*	[26] and references therein	Non-canonical intrinsic
ISSau8 Family: ISL3	*S. aureus* MRSA252	[26] and references therein	NT
ISSau3 Family: IS1182	*S. aureus* MRSA252,<br>*S. aureus* MSSA476	[26] and references therein	NT-FD
REP	*P. aeruginosa*	[27]	NT
PU/REP/BIME†<br>p.u./PU – palindromic unit<br>REP – repetitive extragenic palindromes<br>BIME – bacterial interspersed mosaic element	UPEC, ETEC, EPEC,<br>*S. enterica,*<br>*K. pneumonia,*<br>*S. flexneri*	[28]; for successive reports and element refined definitions see [18], and references therein	Unclassified element (and NT for *K. pneumoniae*)
ISEc13 Family: IS4	EPEC	[26] and references therein	Unclassified element

* – Alternative names DUS, DNA uptake signal, Uptake signal sequence (USS)
† – This element has multiple variants and multiple names; not all names are listed.
‡ – The references are element oriented. Thus, they often, but not always, relate to the specific organism in which we found them.
§ – For category details see main text. (NT; No ter-peak) – No ter-peak was observed for the motif associated with the element; (FD; Fixed Distance) – The motif associated with the element was located at a fixed distance > 15 nt from the 3′ terminus position, based on the MAD value. Note that multiple motifs may match the elements. In these cases, the element was classified by its best characterized motif.

Supplementary References

1. Bailey, T.L., Boden, M., Buske, F.A., Frith, M., Grant, C.E., Clementi, L., Ren, J., Li, W.W., and Noble, W.S. (2009). MEME SUITE: tools for motif discovery and searching. Nucleic Acids Res *37*, W202–208. 10.1093/nar/gkp335.
2. Tatusova, T., DiCuccio, M., Badretdin, A., Chetvernin, V., Nawrocki, E.P., Zaslavsky, L., Lomsadze, A., Pruitt, K.D., Borodovsky, M., and Ostell, J. (2016). NCBI prokaryotic genome annotation pipeline. Nucleic Acids Res *44*, 6614–6624. 10.1093/nar/gkw569.
3. Benjamini, Y., and Hochberg, Y. (1995). Controlling the false discovery rate: a practical and powerful approach to multiple testing. Journal of the Royal statistical society: series B (Methodological) *57*, 289–300.
4. Danner, D.B., Deich, R.A., Sisco, K.L., and Smith, H.O. (1980). An eleven-base-pair sequence determines the specificity of DNA uptake in Haemophilus transformation. Gene *11*, 311–318. 10.1016/0378-1119(80)90071-2.
5. Smith, H.O., Tomb, J.F., Dougherty, B.A., Fleischmann, R.D., and Venter, J.C. (1995). Frequency and distribution of DNA uptake signal sequences in the Haemophilus influenzae Rd genome. Science *269*, 538–540. 10.1126/science.7542802.
6. Thomson, V.J., Bhattacharjee, M.K., Fine, D.H., Derbyshire, K.M., and Figurski, D.H. (1999). Direct selection of IS903 transposon insertions by use of a broad-host-range vector: isolation of catalase-deficient mutants of Actinobacillus actinomycetemcomitans. J Bacteriol *181*, 7298–7307. 10.1128/jb.181.23.7298-7307.1999.
7. Wang, Y., Goodman, S.D., Redfield, R.J., and Chen, C. (2002). Natural transformation and DNA uptake signal sequences in Actinobacillus actinomycetemcomitans. J Bacteriol *184*, 3442–3449. 10.1128/jb.184.13.3442-3449.2002.
8. Ambur, O.H., Frye, S.A., and Tønjum, T. (2007). New functional identity for the DNA uptake sequence in transformation and its presence in transcriptional terminators. J Bacteriol *189*, 2077–2085. 10.1128/jb.01408-06.
9. Goodman, S.D., and Scocca, J.J. (1988). Identification and arrangement of the DNA sequence recognized in specific transformation of Neisseria gonorrhoeae. Proc Natl Acad Sci U S A *85*, 6982–6986. 10.1073/pnas.85.18.6982.
10. Smith, H.O., Gwinn, M.L., and Salzberg, S.L. (1999). DNA uptake signal sequences in naturally transformable bacteria. Res Microbiol *150*, 603–616. 10.1016/s0923-2508(99)00130-8.
11. Correia, F.F., Inouye, S., and Inouye, M. (1986). A 26-base-pair repetitive sequence specific for Neisseria gonorrhoeae and Neisseria meningitidis genomic DNA. J Bacteriol *167*, 1009–1015. 10.1128/jb.167.3.1009-1015.1986.
12. Correia, F.F., Inouye, S., and Inouye, M. (1988). A family of small repeated elements with some transposon-like properties in the genome of Neisseria gonorrhoeae. J Biol Chem *263*, 12194–12198.
13. Liu, S.V., Saunders, N.J., Jeffries, A., and Rest, R.F. (2002). Genome analysis and strain comparison of correia repeats and correia repeat-enclosed elements in pathogenic Neisseria. J Bacteriol *184*, 6163–6173. 10.1128/JB.184.22.6163-6173.2002.
14. Mazzone, M., De Gregorio, E., Lavitola, A., Pagliarulo, C., Alifano, P., and Di Nocera, P.P. (2001). Whole-genome organization and functional properties of miniature DNA insertion sequences conserved in pathogenic Neisseriae. Gene *278*, 211–222. 10.1016/s0378-1119(01)00725-9.

15. Snyder, L.A., Cole, J.A., and Pallen, M.J. (2009). Comparative analysis of two Neisseria gonorrhoeae genome sequences reveals evidence of mobilization of Correia Repeat Enclosed Elements and their role in regulation. BMC Genomics *10*, 70. 10.1186/1471-2164-10-70.
16. Croucher, N.J., Vernikos, G.S., Parkhill, J., and Bentley, S.D. (2011). Identification, variation and transcription of pneumococcal repeat sequences. BMC Genomics *12*, 120. 10.1186/1471-2164-12-120.
17. Martin, B., Humbert, O., Camara, M., Guenzi, E., Walker, J., Mitchell, T., Andrew, P., Prudhomme, M., Alloing, G., Hakenbeck, R., and et al. (1992). A highly conserved repeated DNA element located in the chromosome of Streptococcus pneumoniae. Nucleic Acids Res *20*, 3479–3483. 10.1093/nar/20.13.3479.
18. Bachellier, S., Clement, J.M., and Hofnung, M. (1999). Short palindromic repetitive DNA elements in enterobacteria: a survey. Res Microbiol *150*, 627–639. 10.1016/s0923-2508(99)00128-x.
19. De Gregorio, E., Silvestro, G., Venditti, R., Carlomagno, M.S., and Di Nocera, P.P. (2006). Structural organization and functional properties of miniature DNA insertion sequences in yersiniae. J Bacteriol *188*, 7876–7884. 10.1128/jb.00942-06.
20. Cramton, S.E., Schnell, N.F., Gotz, F., and Bruckner, R. (2000). Identification of a new repetitive element in Staphylococcus aureus. Infect Immun *68*, 2344–2348. 10.1128/IAI.68.4.2344-2348.2000.
21. Barker, A., Clark, C.A., and Manning, P.A. (1994). Identification of VCR, a repeated sequence associated with a locus encoding a hemagglutinin in Vibrio cholerae O1. J Bacteriol *176*, 5450–5458. 10.1128/jb.176.17.5450-5458.1994.
22. Gardner, P.P., Barquist, L., Bateman, A., Nawrocki, E.P., and Weinberg, Z. (2011). RNIE: genome-wide prediction of bacterial intrinsic terminators. Nucleic Acids Res *39*, 5845–5852. 10.1093/nar/gkr168.
23. De Gregorio, E., Bertocco, T., Silvestro, G., Carlomagno, M.S., Zarrilli, R., and Di Nocera, P.P. (2009). Structural organization of a complex family of palindromic repeats in Enterococci. FEMS microbiology letters *292*, 7–12. 10.1111/j.1574-6968.2008.01461.x.
24. Petrillo, M., Silvestro, G., Di Nocera, P.P., Boccia, A., and Paolella, G. (2006). Stem-loop structures in prokaryotic genomes. BMC Genomics *7*, 170. 10.1186/1471-2164-7-170.
25. Venditti, R., De Gregorio, E., Silvestro, G., Bertocco, T., Salza, M.F., Zarrilli, R., and Di Nocera, P.P. (2007). A novel class of small repetitive DNA sequences in Enterococcus faecalis. FEMS microbiology letters *271*, 193–201. 10.1111/j.1574-6968.2007.00717.x.
26. Siguier, P., Perochon, J., Lestrade, L., Mahillon, J., and Chandler, M. (2006). ISfinder: the reference centre for bacterial insertion sequences. Nucleic Acids Res *34*, D32–36. 10.1093/nar/gkj014.
27. Tobes, R., and Ramos, J.L. (2005). REP code: defining bacterial identity in extragenic space. Environ Microbiol *7*, 225–228. 10.1111/j.1462-2920.2004.00704.x.
28. Higgins, C.F., Ames, G.F., Barnes, W.M., Clement, J.M., and Hofnung, M. (1982). A novel intercistronic regulatory element of prokaryotic operons. Nature *298*, 760–762. 10.1038/298760a0.
